# Pharmacophore-based virtual screening of commercial databases against β-secretase 1 for drug development against Alzheimer’s disease

**DOI:** 10.3389/fchem.2024.1412349

**Published:** 2024-07-09

**Authors:** Xu Han, Kaibo Guo

**Affiliations:** ^1^ The First Clinical Medical College of Zhejiang Chinese Medical University, Hangzhou, Zhejiang, China; ^2^ Department of Oncology, Affiliated Hangzhou First People’s Hospital, School of Medicine, Westlake University, Hangzhou, China

**Keywords:** pharmacophore-based virtual screening, β-secretase 1, Alzheimer’s disease, databases, crystal structure

## Abstract

β-secretase 1, one of the most important proteins, is an aspartate protease. This membrane-associated protein is used for treating Alzheimer’s disease (AD). Several inhibitors have been pursued against β-secretase 1, but they still have not resulted effectively. Virtual screening based on pharmacophores has been shown to be useful for lead optimization and hit identification in the preliminary phase of developing a new drug. Here, we screen the commercially available databases to find the hits against β-secretase 1 for drug discovery against AD. Virtual screening for 200,000 compounds was done using the database from the Vitas-M Laboratory. The phase screen score was utilized to assess the screened hits. Molecular docking was performed on compounds with phase scores >1.9. According to the study, the 66H ligand of the crystal structure has the maximum performance against β-secretase 1. The redocking of the co-crystal ligand showed that the docked ligand was seamlessly united with the crystal structure. The reference complex had three hydrogen bonds with Asp93, Asp289, and Gly291; one van der Waals interaction with Gly74; and three hydrophobic interactions. After equilibration, the RMSD of the reference compound sustained a value of ∼1.5 Å until 30 ns and then boosted to 2.5 Å. On comparison, the RMSD of the S1 complex steadily increased to ∼2.5 Å at 15 ns, displayed slight aberrations at approximately ∼2.5–3 Å until 80 ns, and then achieved steadiness toward the end of the simulation. The arrangements of proteins stayed condensed during the mockup when bonded to these complexes as stable Rg values showed. Furthermore, the MM/GBSA technique was employed to analyze both compounds’ total binding free energies (ΔGtotal). Our research study provides a new understanding of using 66H as anti-β-secretase 1 for drug development against AD.

## 1 Introduction

Neurodegenerative disorders (NDDs) are characterized by the gradual deterioration and loss of specific groups of neurons, primarily those affiliated with the central nervous system (Behl et al., 2021). Alzheimer’s disease (AD) causes neurodegenerative changes characterized by an advanced, irreversible, and subtle weakening of cognitive function, including memory loss and various cognitive impairments (Yusufzai et al., 2018). AD is a widespread and frequently encountered type of dementia (Bogdanovic et al., 2020), associated with decreased memory and cognition (Marelli et al., 2020; Wilson et al., 2012). A sixth leading cause of death is in the geriatric population (Gaugler et al., 2019). The progression of AD comprises three primary aspects. First, a lack of cholinergic transmission results from losing cholinergic neurons. Second, the buildup of extracellular residues of *β*-amyloid protein occurs, owing to the catalytic action of β-secretase 1 (BACE1). Last, neurofibrillary bundles comprise a tau protein in the phosphorylated form (Falco et al., 2016; Selkoe and Hardy, 2016; Gaugler et al., 2019). The development of extracellular residues of the *β*-amyloid peptide and the buildup of unsolvable plaques in neurons are central to the amyloid hypothesis, which connects AD to this pathophysiological process. This process starts the transmembrane protein (amyloid precursor protein-APP) breakdown by the enzyme BACE1. Another enzyme, *γ*-secretase, terminates this cleavage process and generates the *β*-amyloid peptide (A*β*), aggregating into oligomers. These oligomers form plaques that accumulate in many regions of the brain, mainly in neurons located in the cortex entorhinal, hippocampus, basal nucleus, and associative cortex (Sabbah and Zhong, 2016). A recently FDA-approved drug called Aduhelm^©^ (aducanumab) is among the few medications utilized to address the amyloid hypothesis (FDA Grants Accelerated Approval for Alzheimer’s Drug | FDA). This drug functions as a monoclonal antibody specifically designed to target combined forms of amyloid beta agglomerates, thereby reducing the accumulation of extracellular deposits of the *β*-amyloid peptide. BACE1 is one of the most essential membrane-associated aspartate protease proteins focused on treating AD (Ghosh et al., 2012; Kandalepas and Vassar, 2012; Yan and Vassar, 2014). Beta-amyloid peptide (Aβ) development in AD can be terminated by inhibiting BACE1 (Boutajangout et al., 2011; Kwak et al., 2011; Yan and Vassar, 2014). The formation of BACE1 inhibitors, which is followed for many years, has still not been established as an effective treatment method. However, constant improvement in this sphere has led to the formation of inhibitors that display widespread activity, from nano to micromolar. Consequently, evolving inhibitors for BACE1 have been an effective curative approach for AD drug discovery. A pharmacophore is an organization of structural elements and molecular features related to biological activity (Wermuth, 2006). Lately, this phrase has become one of the most well-known icons in the discovery of drugs. As an esteemed tool for drug planning, virtual pharmacophore screening has been established as valuable for lead optimization and hit identification in the preliminary phase of the novel drug development (Gautam et al., 2023). The benefit of this method is that virtually, most compounds can be screened for hit identification. Points in 3D space usually signify pharmacophore features. A feature of pharmacophores could be composed of functional groups, such as the hydrogen bond acceptor (HBA), hydrogen bond donor (HBD), anions, cations, hydrophobic area (Hyp), and aromatics (Dror et al., 2004; Hou et al., 2006). Cautiousness should be employed while controlling the structural flexibility for generating pharmacophores where the active conformation of particles is hypothesized.

The study aimed to address the urgent need for effective treatments for AD, characterized by the accumulation of amyloid-beta plaques in the brain. BACE1, being a crucial protein involved in the production of amyloid-beta, represents a promising therapeutic target for AD. However, despite extensive efforts, existing BACE1 inhibitors have not been sufficiently effective in clinical trials.

The method of receptor or ligand-based pharmacophore virtual screening includes a diversity of chronological computational steps: target identification, preparation of database, pharmacophore model creation, 3D screening, and arrangement of complexes for the final confirmation of biological activity (Köppen, 2009; Liu et al., 2023). Virtual screening provides a profitable, time-saving method in the novel lead compound search (Muhammed and Esin, 2021). Screening virtually is an obligatory part of the drug discovery pipeline and a vital procedure for finding hits or chemical probes (Kumar and Zhang, 2015; Leung and Ma, 2015). In this study, we have targeted the commercially available databases to discover the pharmacophore-based virtual screening against BACE1 for drug discovery against AD.

The study offers medicinal chemists, biochemists, and pharmacologists a promising avenue for advancing AD therapeutics through the identification and characterization of a novel compound, 66H, targeting BACE1. Through virtual screening and molecular docking, 66H emerged as a lead candidate, with subsequent molecular dynamics simulations confirming its stable binding to BACE1. The study also explored the key molecular interactions and assessed the compound’s binding affinity using MM/GBSA analysis, providing crucial insights for further medicinal chemistry optimization and biochemical validation.

## 2 Methodology

### 2.1 Pharmacophore hypothesis development

Protein Data Bank (https://www.rcsb.org/) was used for retrieving the crystal structures of the ΒΑCE1 protein. One of the studies suggested the co-crystal ligands’ activity against ΒΑCE1 was taken into consideration (Stamford and Strickland, 2013; Egbertson et al., 2015; Gupta et al., 2020). The pharmacophore model, which was receptor–ligand-based, was developed according to the inhibitor with the highest activity against BACE1. The Schrödinger phase tool acquired the pharmacophore hypothesis (Dixon et al., 2006). The protein-binding pocket and ligand sites were targeted for building the hypothesis. Moreover, the receptor was prepared by following the steps in Section 2.4 before the hypothesis was developed.

### 2.2 Preparation and virtual screening of database

The VITAS-M Laboratory database comprised 1.4 million compounds, from which 0.2 million complexes were selected. (Dixon et al., 2006), transferred, and arranged through phases. Ten conformers were produced for each ligand to expand the search for chemical space. Epik generated diverse likely states at pH 7, while the tautomeric states (Shelley et al., 2007), having high-energy, were eliminated from the database. Then, virtual screening was initiated from the prepared database, agreeing to the developed hypothesis. The phase screen score was used to assess the screened hits according to the mixture of volume score, RMSD, and site matching. Phase scores >1.9 were identified for the molecular docking studies.

### 2.3 ADMET and drug-likeness

The selective commercial complexes were subjected to absorption, metabolism, distribution, excretion, and toxicity (ADMET) in the QikProp module of Maestro, and ADME (http://www.swissadme.ch) and ADMETlab 2.0 (https://admetmesh.scbdd.com/) to evaluate ADMET and drug-likeness parameters. Compounds which passed the Lipinski’s rule of five and toxicity parameters were considered for further analysis. Different software tools, such as QikProp, ADME, and ADMET, have been employed to comprehensively assess the pharmacokinetic and pharmacodynamics properties (ADMET) of the identified compound. QikProp is known for its ability to predict a wide range of physicochemical properties. ADME specializes in offering insights into the compound’s bioavailability and metabolic stability. ADMET mainly focuses on predicting potential adverse effects and toxicity profiles.

### 2.4 Molecular docking

The β-secretase 1 (PDB ID: 5HU0) crystal structure was prepared in Maestro (Madhavi Sastry et al., 2013). The receptor was preprocessed by introducing hydrogens and charges, eliminating water and setting the residue side-chain atoms. The redundant sequences were eliminated, while the tautomeric states at pH 7 were produced (Sadeer et al., 2019), employing PropKa. The protein receptor was further organized and minimized by OPLS_2005 force field (Shivakumar et al., 2012). The framework was created by choosing the site-specific crystal ligand to complete docking. To unstiffen the activity of non-polarized receptor slices, the radii of the receptor atom, i.e., the van der Waals, were graduated to 1.0, with the partial charge limit set to 0.25. The coordinates X, Y, and Z results were 23.55, 10.39, and 21.58, respectively. After grid creation, the ligands were primed by the LigPrep tool of Maestro before docking (Matsuoka et al., 2017). Diverse ionization shapes were produced at pH 7 by employing Epik (Shelley et al., 2007). The isomers of complexes with definite chirality are produced using the OPLS_2005 force field. A glide docking tool was used to stop ligands’ arrangement to the set protein receptor, and the binding positions were evaluated according to the glide score.

### 2.5 Molecular dynamics (MD) simulation

The best binding poses of the particular hits and reference complex with protein were subjected to 100 ns using NAMD (Phillips et al., 2020) and VMD (Humphrey et al., 1996) to discover their strength. As an initial phase, the preliminary records necessary to execute the simulation were arranged through the elements (Case et al., 2021) of AmberTools 21. The components of an antechamber created the parameters of the conjugate solution, while the LEAP program (Case et al., 2005) added the lost hydrogen atoms in the protein arrangements. TIP3P water particles were introduced to the structures (Jorgensen and Chandrasekhar, 1983) in a periodic box after the parameterization of 10 Å and then defused by adding sodium cations. The energy conflicts were eliminated by minimizing the technique using the ff14SB force field for GAFF ligands and protein (Duan et al., 2003). After depreciation, solvation was equilibrated for 10,000 steps, which was trailed by temperature balancing at 200, 250, and 300 K. The concluding equilibrated procedures were then exposed to a 100-ns production run, and the trajectories were saved at every 2 ps for the evaluation. The Bio3D package of R was used to calculate the MD trajectories (Grant et al., 2021).

## 3 Results

### 3.1 Alignment of protein structures

Protein Data Bank was used to retrieve the crystal structures of the ΒΑCE1 protein. The literature was searched for the IC_50_ cut-offs of the co-crystal ligands. As evident, the ligand of co-crystal 66H has shown maximum performance against the protease protein between the selected ligands; thus, it was chosen to study further. The IDs of PDB crystals and the IC_50_ cut-offs are presented in Table 1.

**TABLE 1:** IDs of PDB, ligands, and the chemical mechanism of a co-crystal ligand against the BACE1 protein.

PDB	PDB ligand	Resolution	IC_50_
1TQF	32P	1.80	1,400 nM
5HU0	66H	1.83	1.7 nM
1W51	L01	2.55	200 nM
1XS7	MMI	2.80	3,900 nM
2B8L	5HA	1.70	35 nM
2FDP	FRP	2.50	26 nM
2HIZ	LIJ	2.50	67 nM
2HM1	LIQ	2.20	3 nM
2IQG	F2I	1.70	130 nM
2IRZ	I02	1.80	12 nM
2IS0	103	2.20	10 nM
2NTR	L00	1.80	16,500 nM
2OAH	QIN	1.80	34 nM
2P8H	MY9	1.80	11 nM
2P83	MR0	2.50	11 M

### 3.2 Generation of the receptor-based pharmacophore

**FIGURE 1:**
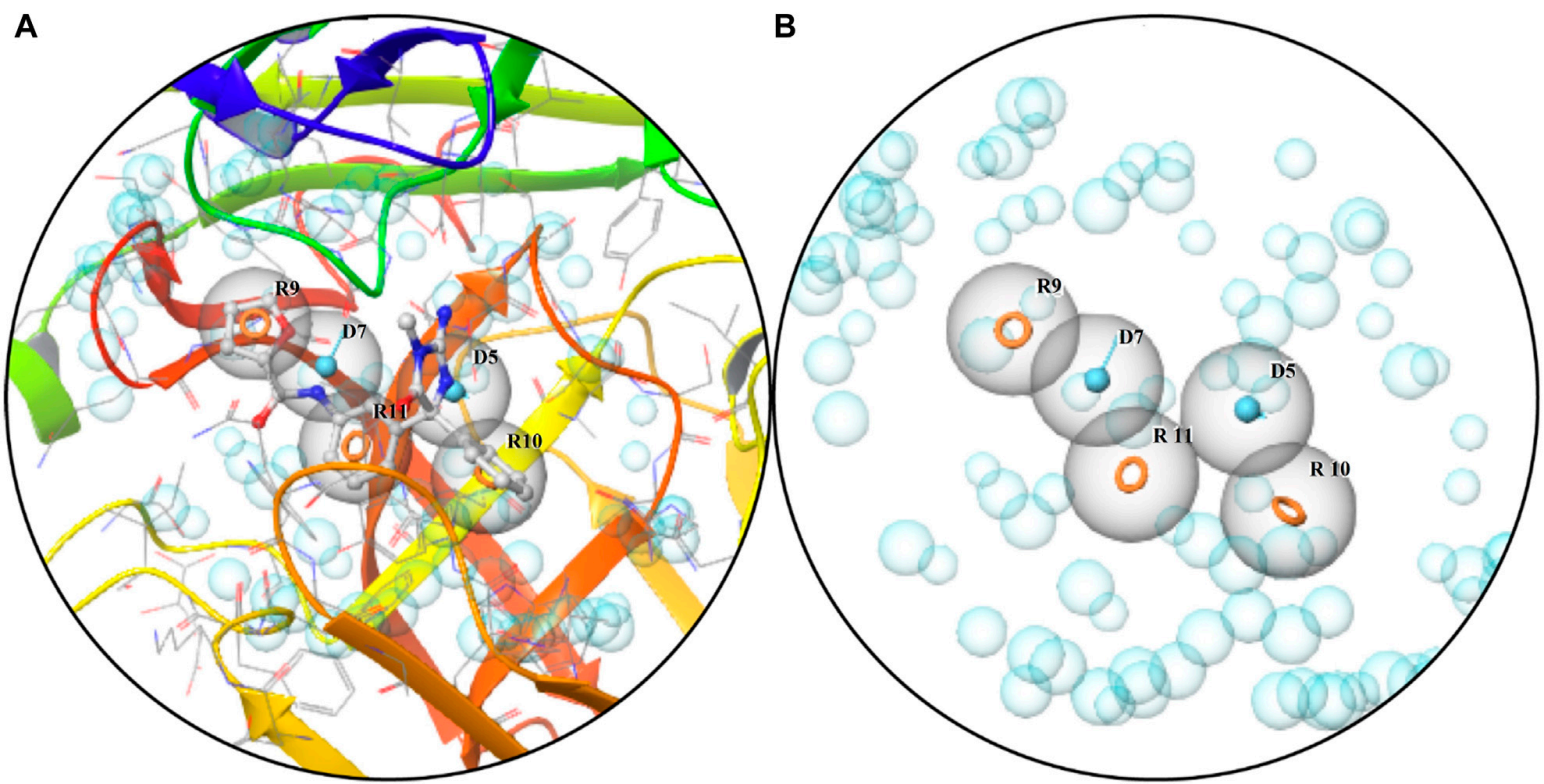
Pharmacophore: **(A)** characteristics of the binding pocket and **(B)** pharmacophore hypothesis and the binding pocket cavity.

**FIGURE 2:**
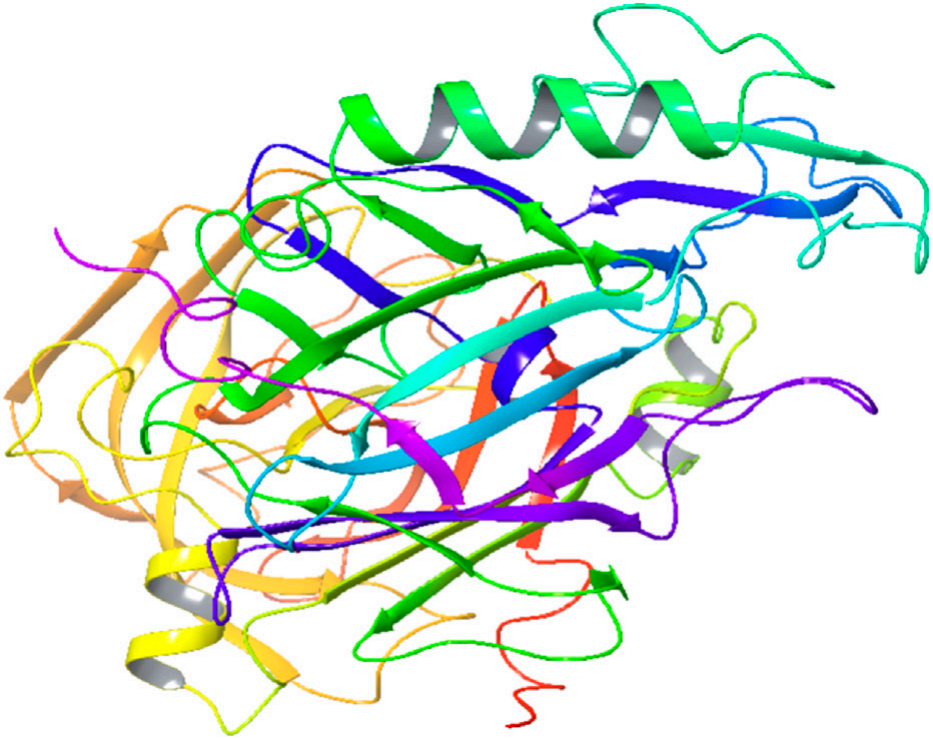
Crystal structure of β-secretase 1.

A five-feature pharmacophore model was created by choosing ligand sites and pocket residues with specific binding. The pharmacophore hypothesis comprised features such as R9, R10, R11, D9, and D7, along with their coordinates in the structure of protein (Table 2; Figure 1A). The descriptions of the binding pocket’s cavity are witnessed in Figure 2B.

**TABLE 2:** Coordinates and scores for the features within the hypothesis of pharmacophores.

Rank	Feature label	X	Y	Z	Score
1	R10	18.97	10.26	20.34	−1.08
2	R9	29.45	9.49	22.30	−0.89
3	R11	23.97	10.76	19.82	−0.84
4	D7	26.38	9.48	21.75	−0.51
5	D5	21.85	8.43	22.17	−0.32

### 3.3 Virtual screening

The database of Vitas-M Laboratory library was utilized to screen the hypothesis of pharmacophores virtually. At least four features have been selected to identify a complex as a hit. The final ranking of hits from the screening, contributing to the phase fitness score, was determined by comprising vector arrangements, volume scores, and matching RMSD site. The range of vector scores is from −1 to 1, where greater scores indicate improved alignment. On the other hand, the volume scores ranging from 0 to 1 indicate a higher overlap among the reference and aligned ligand levels with higher scores. It is determined as the overlaying of both the volumes of the ligand divided by the total volume of both ligands. The score is zero if no reference ligand is present. A cutoff score of 1.9 during the phase screen was chosen to identify the potential hits above this threshold (Table 3). The structures of 84 hits have been provided in Supplementary Figures S1–S84.

**TABLE 3:** Hit alignment and phase screen scores of a pharmacophore model.

S. No.	Compound ID	Vector score	Volume score	Align score	Phase score
1	STK081237	0.892	0.691	0.31	2.08
2	STK280616	0.934	0.643	0.453	2.017
3	STK057995	0.934	0.618	0.453	1.992
4	STK408850	0.934	0.617	0.453	1.99
5	STK067256	0.932	0.629	0.482	1.988
6	STK100429	0.942	0.604	0.466	1.979
7	STK362117	0.863	0.606	0.275	1.977
8	STK387431	0.934	0.593	0.453	1.967
9	STK046443	0.735	0.555	0.392	1.963
10	STK136267	0.948	0.587	0.481	1.962
11	STK386021	0.934	0.589	0.466	1.958
12	STK137950	0.938	0.58	0.467	1.952
13	STK385466	0.941	0.575	0.466	1.95
14	STK385674	0.941	0.575	0.466	1.95
15	STK072483	0.941	0.572	0.466	1.947
16	STK154114	0.939	0.574	0.466	1.947
17	STK012551	0.941	0.572	0.466	1.947
18	STK131655	0.923	0.595	0.481	1.945
19	STK154089	0.935	0.576	0.467	1.944
20	STK129297	0.934	0.576	0.466	1.944
21	STK129615	0.938	0.572	0.467	1.944
22	STK090091	0.941	0.567	0.466	1.941
23	STK129509	0.923	0.588	0.482	1.938
24	STK129571	0.913	0.587	0.467	1.933
25	STK113693	0.883	0.571	0.365	1.931
26	STK222598	0.935	0.575	0.496	1.931
27	STK155936	0.941	0.611	0.58	1.93
28	STK222602	0.935	0.575	0.496	1.93
29	STK409019	0.939	0.592	0.54	1.93
30	STK137196	0.934	0.571	0.486	1.929
31	STK154090	0.938	0.558	0.467	1.929
32	STK130675	0.913	0.579	0.467	1.926
33	STK007472	0.923	0.569	0.469	1.925
34	STK068025	0.673	0.589	0.404	1.925
35	STK073398	0.93	0.559	0.463	1.924
36	STK075179	0.913	0.578	0.467	1.924
37	STK386018	0.913	0.578	0.467	1.924
38	STK130489	0.924	0.588	0.515	1.923
39	STK132568	0.96	0.585	0.581	1.923
40	STK401920	0.934	0.59	0.54	1.923
41	STK135071	0.913	0.576	0.467	1.922
42	STK137123	0.913	0.575	0.467	1.922
43	STK048780	0.913	0.574	0.467	1.921
44	STK031760	0.913	0.574	0.467	1.921
45	STK039660	0.913	0.574	0.467	1.92
46	STK092252	0.93	0.556	0.466	1.92
47	STK121703	0.935	0.564	0.496	1.919
48	STK325732	0.913	0.573	0.467	1.919
49	STK386010	0.926	0.585	0.52	1.919
50	STK337539	0.934	0.587	0.54	1.919
51	STK324799	0.938	0.577	0.529	1.919
52	STK337540	0.924	0.583	0.515	1.917
53	STK408865	0.939	0.581	0.542	1.917
54	STK154061	0.913	0.57	0.467	1.917
55	STK409037	0.924	0.581	0.515	1.916
56	STK409038	0.924	0.581	0.515	1.916
57	STK000255	0.913	0.569	0.467	1.915
58	STK154110	0.938	0.543	0.467	1.914
59	STK020405	0.928	0.575	0.517	1.913
60	STK401922	0.924	0.577	0.515	1.912
61	STK386029	0.913	0.567	0.47	1.912
62	STK154130	0.923	0.555	0.466	1.912
63	STK036626	0.924	0.577	0.515	1.912
64	STK097228	0.858	0.584	0.386	1.91
65	STK100419	0.924	0.575	0.515	1.91
66	STK045387	0.913	0.563	0.467	1.91
67	STK075062	0.931	0.54	0.456	1.909
68	STK122203	0.92	0.578	0.515	1.909
69	STK013762	0.909	0.568	0.47	1.909
70	STK129898	0.913	0.562	0.467	1.909
71	STK053591	0.93	0.539	0.456	1.908
72	STK188417	0.913	0.561	0.467	1.907
73	STK085958	0.913	0.56	0.467	1.906
74	STK061013	0.923	0.55	0.469	1.905
75	STK062148	0.913	0.559	0.467	1.905
76	STK081664	0.913	0.558	0.467	1.905
77	STK324798	0.929	0.566	0.517	1.904
78	STK012081	0.913	0.558	0.467	1.904
79	STK346841	0.953	0.595	0.622	1.904
80	STK044786	0.924	0.569	0.515	1.904
81	STK138023	0.931	0.562	0.52	1.901
82	STK138208	0.931	0.562	0.52	1.901
83	STK386019	0.909	0.559	0.468	1.901
84	STK133249	0.96	0.563	0.58	1.9

### 3.4 β-secretase 1 structure and sequence analysis

The sequence of the BACE1 precursor (P56817) was acquired from UniProt.

*Physiochemical features:* The physiochemical features of the sequences of the BACE1 precursor were determined by ProtParam. The amino acid profile of the BACE1 precursor showed 9.8% residues of leucine along with 8.6% and 7.4% residues of glycine and valine, respectively. The molecular weight was approximately 55,763.79. There were 42 positively charged arginine and lysine residues along with the 55 negatively charged aspartate and glutamic acid residues. The isoelectric point (PI) value of 5.31 specifies that the protein is slightly acidic, whereas the instability index of 44.23 shows that it is somewhat unstable. This instability was predicted due to the existence of certain dipeptides that are lacking from steady proteins. A greater aliphatic index (88.14) revealed that the protein was mildly thermostable, while a lesser GRAVY score (−0.064) implied that the protein may interact well with water. The extinction coefficient is 85,425 M^-1^cm^-1^ at 280 nm, similar to cysteine, tryptophan, and tyrosine concentrations. To calculate protein–ligand and protein–protein interactions in the solution, this coefficient is valuable.

### 3.5 ADMET

The computational tools QikProp, SwissADME, and ADMETlab 2.0 were used to make predictions for a variety of physiochemical (Supplementary Table S1), medicinal chemistry (Supplementary Table S2), absorption, distribution (Supplementary Table S3), metabolism, excretion (Supplementary Table S4), and toxicity parameters (Supplementary Table S5) for a total of 84 distinct chemicals. As a result, ligands with significant pharmacokinetic features fall within acceptable ranges when using ADMET analysis. The ADMET properties show that all compounds were found to have good pharmacokinetic characteristics and no notable side effects. It was also thought that the potential for medical use was positive.

### 3.6 Molecular docking

**FIGURE 3:**
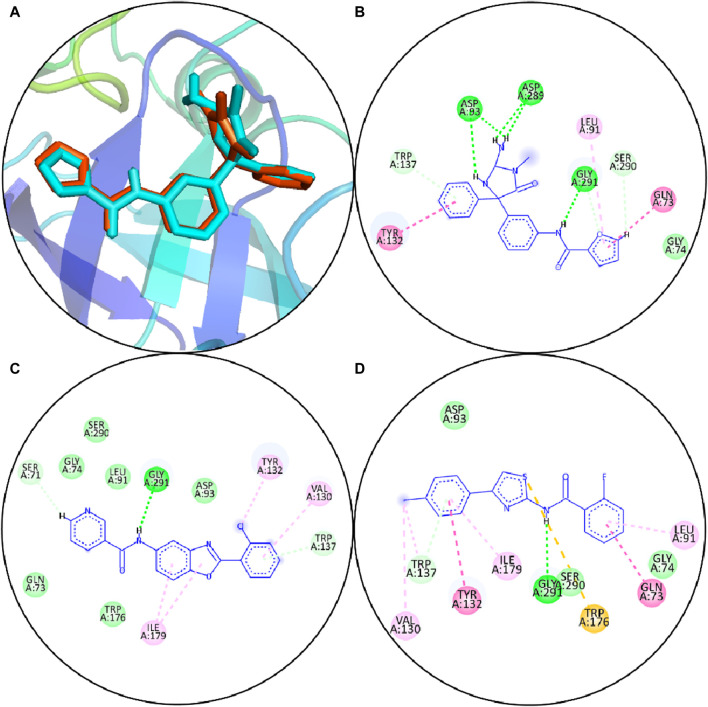
**(A)** Redocking of the reference compound. Orange sticks show the reference pose, and cyan sticks show the docked pose. **(B)** Molecular interactions of reference compounds. **(C)** Molecular interfaces of S1. **(D)** Molecular interactions of S2. Green lines display the hydrogen bonds; light green shows the van der Waals interactions; magenta lines show hydrophobic interactions; purple lines show pi–sigma bonds, and pi–sulfur interactions are shown by orange lines.

The BACE1 receptor was docked by the hits employing the typical precise procedure of the glide tool. Before the docking of screened hits with protein, the effectiveness of this procedure was measured by the redocking of the co-crystal ligand. The redocking of the co-crystal ligand showed that the docked ligand is aligned with the crystal structure (Figure 3A). The docked hits were compared with the reference ligand, and two hits were selected for further analysis. The selected hits with the glide scores are specified in Table 4. The connections of the molecules for the chosen hits were analyzed and compared with the reference compound. The reference complex made three hydrogen bonds with Gly291, Asp93, and Asp289; one van der Waals interaction with Gly74; and three hydrophobic interactions (Figure 3B).

**TABLE 4:** Docking details of the selected and reference complexes.

S No.	Hit ID	Compound ID	Glide score
1	References	66H	−7.019
2	S1	STK346841	−7.73
3	S2	STK122203	−7.67

**FIGURE 4:**
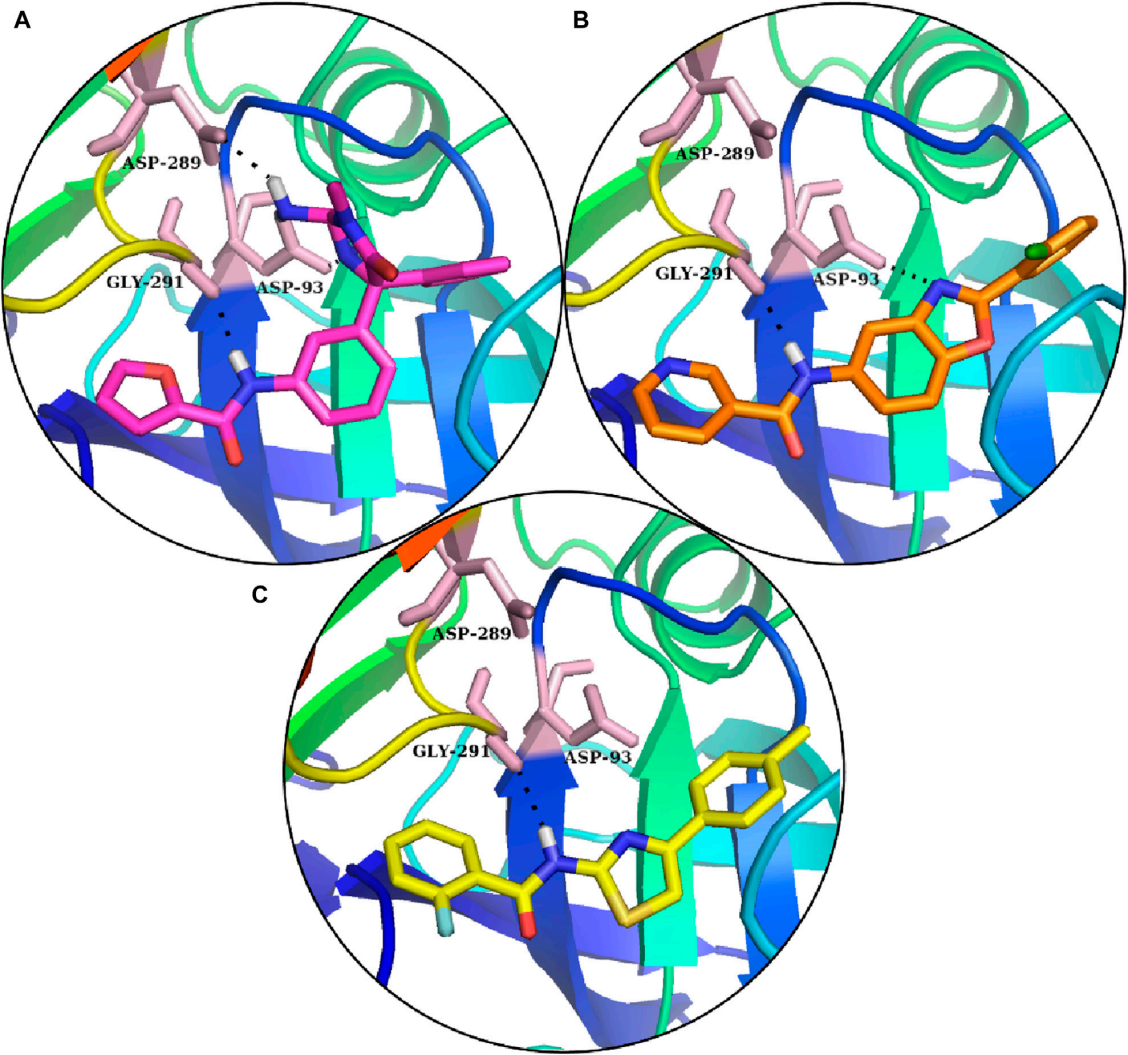
Reference’s plausible binding modes and selected compounds are represented with the sticks in the binding pocket of BACE1. **(A)** Reference compounds: **(B)** S1 hit. **(C)** S2 hit.

In comparison, S1 made one hydrogen bond with Gly291 and five van der Waals interactions with Gln73, Gly74, Leu91, Trp176, and Ser290. Moreover, the reference complex had three hydrophobic interfaces (Figure 3C). S2 also made one hydrogen bond with Gly291; three van der Waals interactions with Gly74, Asp93, and Ser290; and five hydrophobic interactions (Figure 3D). The plausible binding modes of selected docked hits were also analyzed (Figures 4A–C).

### 3.7 MD simulation

**FIGURE 5:**
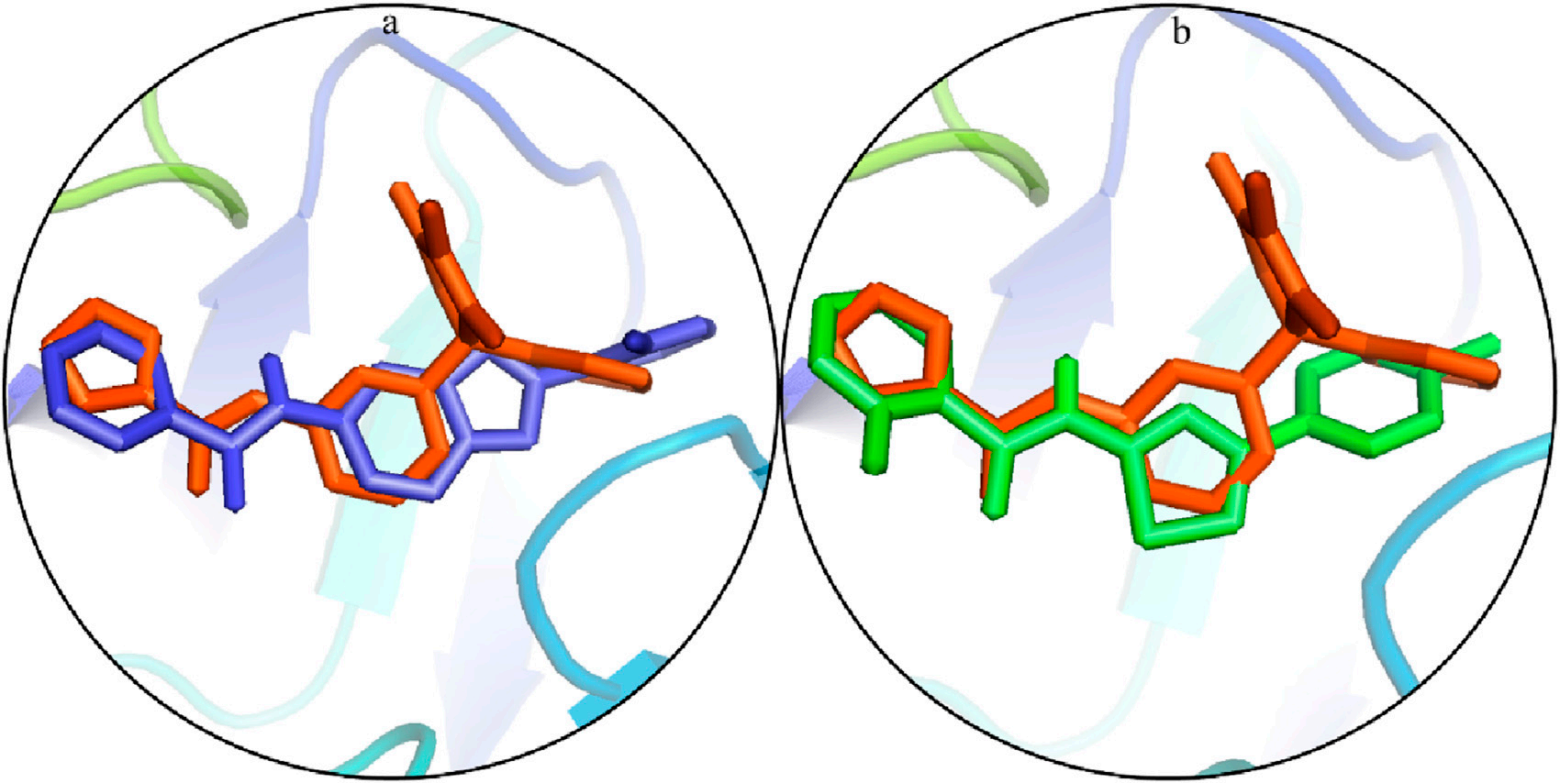
Potential binding configurations of the ligand with the crystal structure: **(A)** S1 is illustrated using blue sticks. **(B)** S2 is depicted using green sticks.

The docked poses of the selected hit ligands were superposed on the co-crystal ligand, as shown in Figures 5A, B, and then subjected to MD simulation for the protein–ligand stability analysis.

**FIGURE 6:**
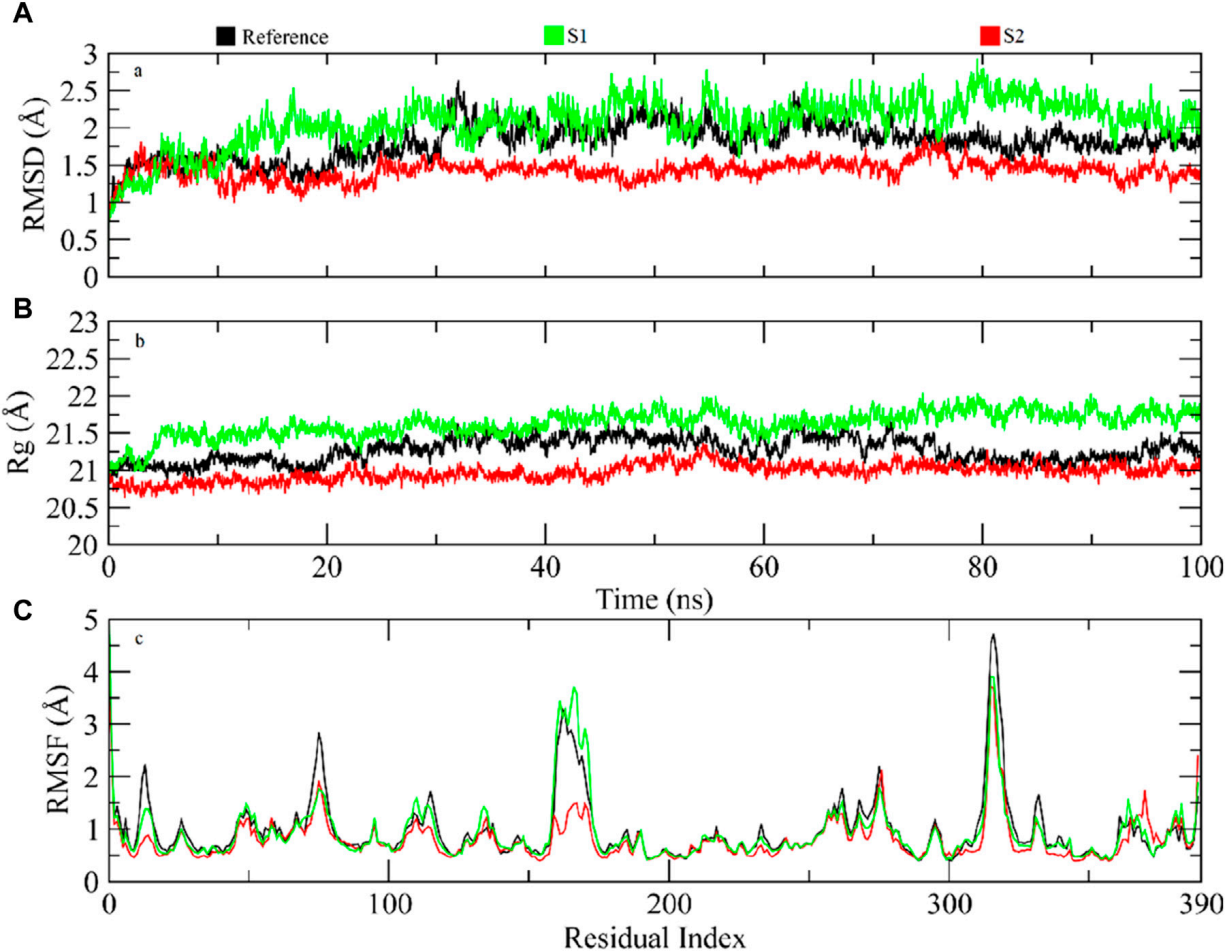
**(A)** RMSD profiles for protein and ligand simulation over 100 ns. **(B)** Analysis of Rg for the density of the protein structure. **(C)** Evaluating residual variations in the protein structure during simulations by RMSF values.

The significant molecules of the protein structure complexed with the reference compound, and hit root mean square deviation (RMSD) values were calculated from the trajectories to assess the steadiness of the protein–ligand compound (Sargsyan et al., 2017). It was witnessed that at 5 ns, all compounds were balanced (Figure 6A). After balancing, the deviation of the reference compound upheld a value of ∼1.5 Å near 30 ns and then enhanced to 2.5 Å. Passing the 30-ns milestone, the RMSD stayed at approximately∼ 1.75–2 Å until the end. On comparison, the RMSD of the S1 complex slowly amplified to ∼2.5 Å near 15 ns, showed slight deviations at approximately ∼2.5–3 Å near 80 ns, and then achieved steadiness toward the end of the simulation. The RMSD values of the S2 complex were approximately ∼1.25–1.5 Å during the simulation. The protein structure’s physical density, when bound to hit and reference complexes, was evaluated by analyzing the radius of gyration (Lobanov et al.). Lesser values of Rg indicated structural solidity, while greater Rg values meant structural deformities throughout the simulation. The Rg profiles for the compounds indicated that Rg values were kept within the range of approximately 20.75–21.5 Å after a 5-ns equilibration period. The Rg value for the S1 complex consistently remained near 21.5 Å during the simulation, whereas the Rg values for the S2 complex remained at approximately 20.75 Å. These stable Rg scores imply that the arrangements of proteins stayed compressed throughout the simulation in the presence of these compounds (refer to Figure 6B). When bound to these ligands, the dynamic behavior of protein restudies was calculated by the root mean square fluctuations (RMSFs) (Martínez, 2015). The RMSF scores of protein deposits fluctuate less than 1 Å throughout the simulation period, excluding the loop regions (Figure 6C). The RMSF figure indicated that the protein residues were rigid and did not show major fluctuations during simulation, suggesting the steadiness of the protein–ligand complex. The RMSF value of the loop residues extended to the highest value of ∼5 Å in the reference compound–complex.

The RMSF graph reveals some regions with high fluctuations within the protein structure, indicating areas of increased flexibility. Specifically, residues around index 100, between indices 200–210, and near index 300 exhibit significant peaks, with the peak around residue 300 being the most pronounced. Additionally, there is also a noticeable flexibility near residue 390. These fluctuations suggest that these particular residues are more dynamic, potentially due to specific interactions in the protein’s structure.

### 3.8 Molecular mechanics/generalized born surface area

**FIGURE 7:**
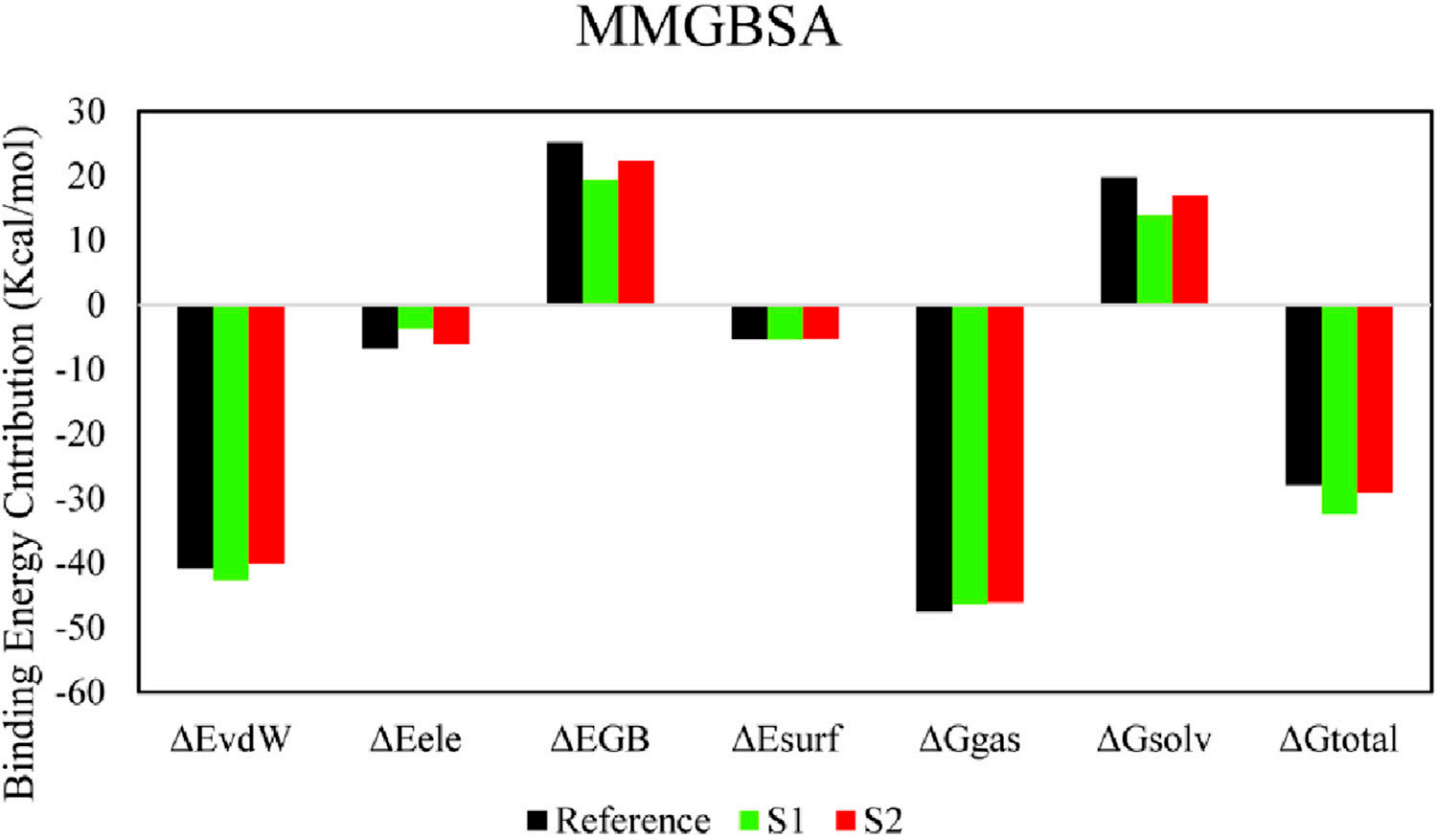
Role of individual binding energy components within the overall binding free energy.

The molecular mechanics/generalized born surface area (MM/GBSA) system helped analyze both complexes’ total binding free energy (ΔGtotal). The result is typically used to evaluate the strength of the ligand–protein compound (Du et al., 2011). The lower ΔG_total_ values specify that the compound is steadier and conversed. It was calculated as a sum of the ligand–protein compound and the difference of protein and its ligands’ free energies. The total binding free energy assessed utilizing the MM/GBSA method is the result of the input of several protein–ligand interfaces such as electrostatic energy (ΔE_ele_), van der Waals energy (ΔE_vdW_), and electrostatic contribution to solvation-free energy by generalized born (ΔG_GB_). The total binding free energies are presented in Table 5. The ΔE_vdW_ role of the S1 compound was more than that of the reference and S2 complexes. At the same time, the electrostatic contribution was more in the reference complex. The GB contribution showed that the reference has a higher GB value than the hits. Both hits’ total binding free energies were more than those of the reference compound, as shown in the table. The total binding free energy and its influence on each energy module are shown in Figure 7.

**TABLE 5:** MM/GBSA module and the binding free energies.

Energy component	Reference	S1	S2
ΔE_vdW_	−40.86 ± 0.63	−42.74 ± 0.47	−40.03 ± 0.35
ΔE_ele_	−6.76 ± 0.68	−3.71 ± 0.21	−6.08 ± 0.31
ΔE_GB_	25.1 ± 0.82	19.42 ± 0.24	22.26 ± 0.28
ΔE_surf_	−5.37 ± 0.07	−5.51 ± 0.03	−5.29 ± 0.02
ΔG_gas_	−47.63 ± 1.07	−46.45 ± 0.49	−46.11 ± 0.34
ΔG_solv_	19.73 ± 0.76	13.91 ± 0.22	16.96 ± 0.28
ΔG_total_	−27.9 ± 0.55	−32.54 ± 0.44	−29.14 ± 0.33

**FIGURE 8:**
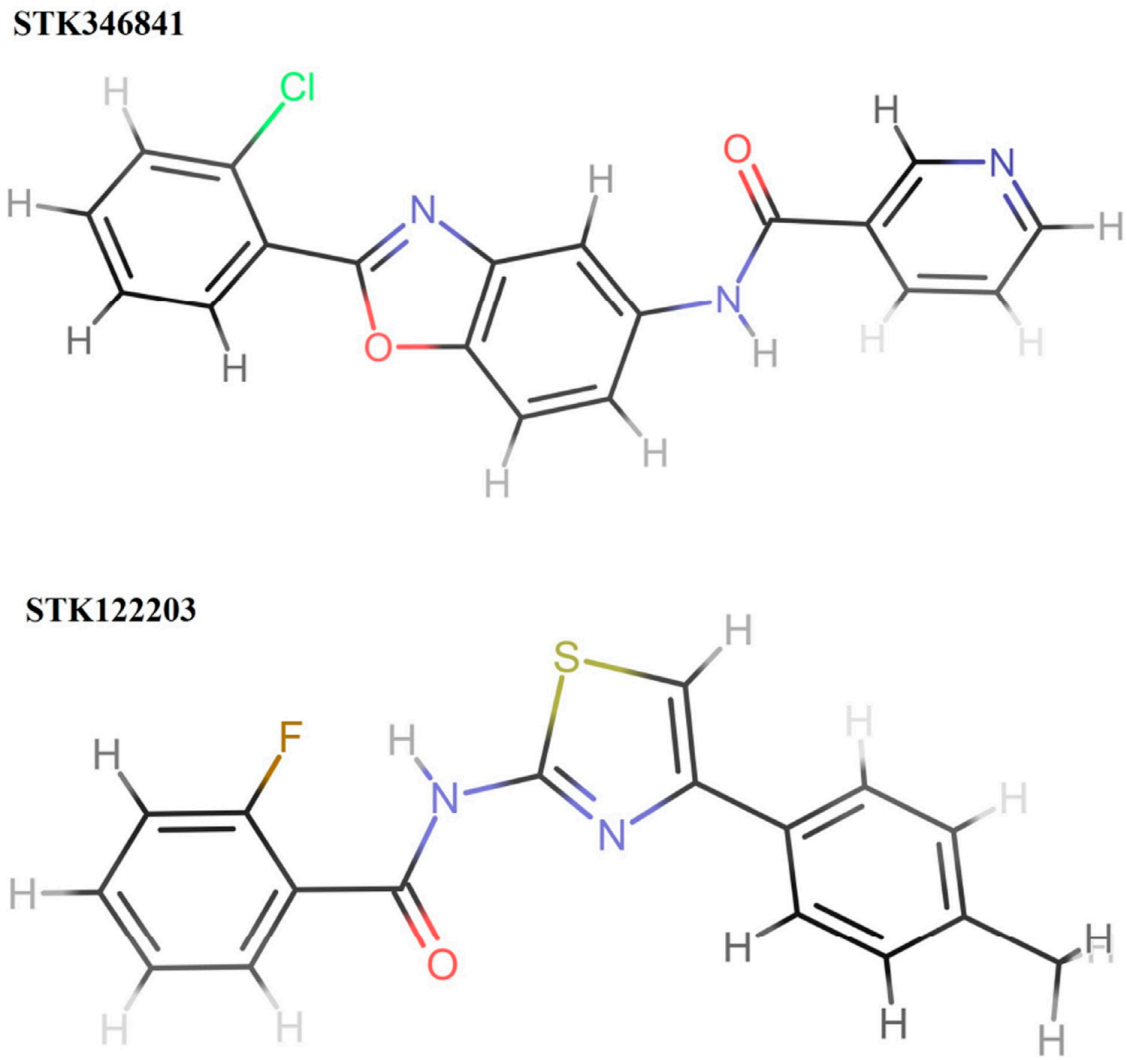
Structures of two hits (STK346841 and STK122203). STK346841 (IUPAC: N-[2-(2-chlorophenyl)-1,3-benzoxazol-5-yl]pyridine-3-carboxamide) has a chlorine (Cl) atom and an imidazole ring as substituents. STK122203 (IUPAC: 2-fluoro-N-[4-(4-methylphenyl)-1,3-thiazol-2-yl]benzamide) features a fluorine (F) atom and a thiazole ring (a ring containing both sulfur and nitrogen).

The structural assessment of STK346841 and STK122203 shows that both have feature benzene rings as foundational elements, characteristic of many aromatic compounds (Figure 8). Each structure also incorporates a heterocyclic ring; specifically, a pyridine ring is present in both, indicating a common structural motif where a nitrogen atom is integrated into a six-membered aromatic ring. This shared feature suggests a similarity in some aspects of their chemical reactivity and possible applications.

**FIGURE 9:**
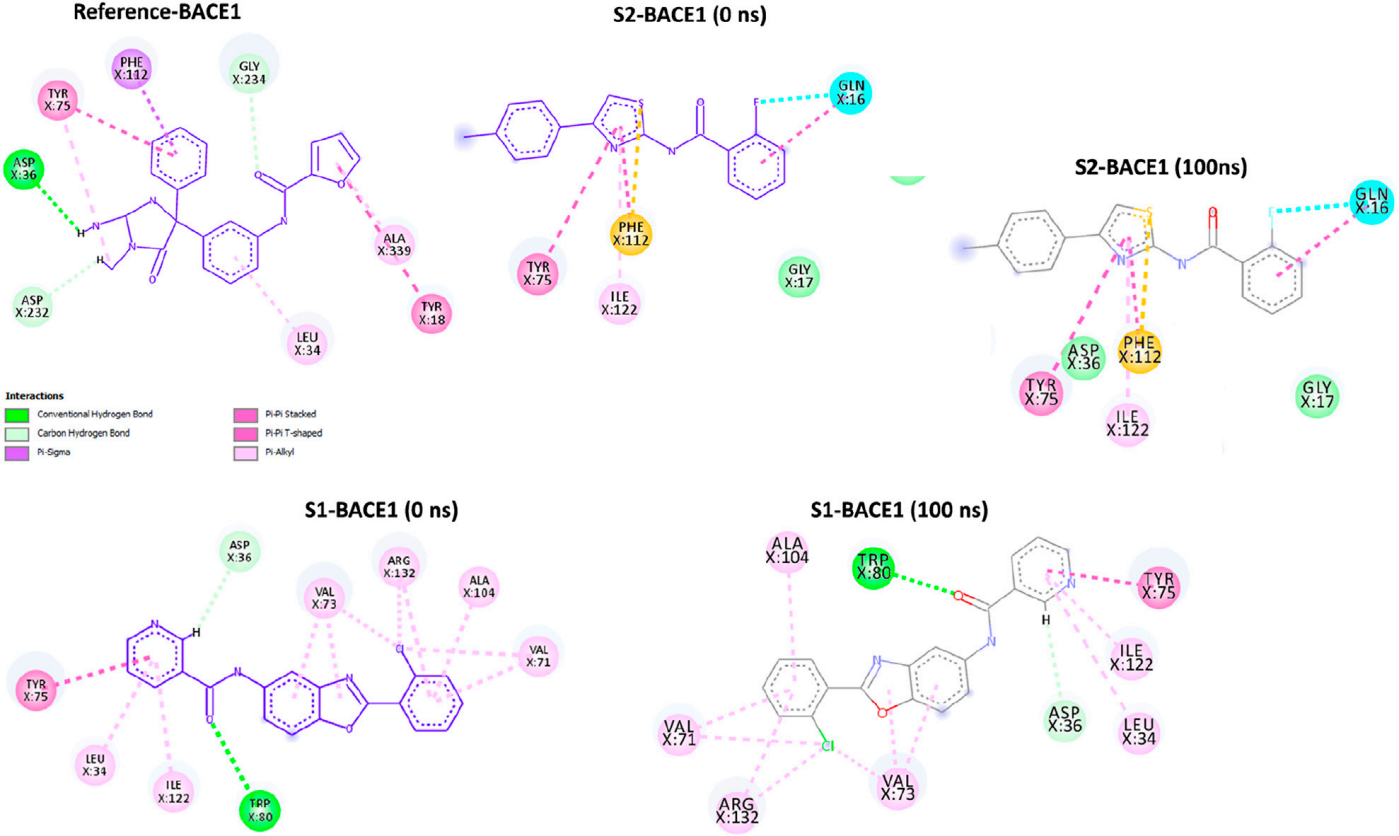
Post-simulation comparison of BAC1 interactions with references S1 and S2.

The post-simulation interaction analysis was performed at 0 ns and 100 ns (Figure 9). There was no significant difference at 0-ns and 100-ns snap shots. All three ligands (references: BACE1, S1-BACE1, and S2-BACE1) show a pi–pi stacked interaction with Tyr75, indicating a critical role of this residue in stabilizing the ligand through aromatic interactions. Similarly, both references BAC1 and S1-BAC1 exhibit pi–alkyl interactions with Leu34, suggesting that hydrophobic interactions with this residue are also important. Moreover, the reference BACE1 has conventional hydrogen bonds with Asp36 and Aap232, which are not present in S2-BACE1. S1-BACE1 retains the hydrogen bond with Asp36 and exhibited one more with Trp80.

## 4 Discussion

The prevalence of neurodegenerative diseases has significantly increased in the medical field over recent years, posing a significant health concern. Various molecular targets are implicated in the pathogenesis of these diseases. These clusters of diseases, including AD and other related disorders such as spinal muscular atrophy (SMA), Parkinson’s disease (PD), Huntington’s disease (HD), spinocerebellar ataxia (SCA), prion disease, and motor neuron diseases (MND), have been reported to affect millions of people worldwide (Dugger and Dickson, 2017). BACE1 is an aspartate protease. This membrane-associated protein treats AD (Ghosh et al., 2012; Kandalepas and Vassar, 2012). The development of beta-amyloid peptide (Aβ) in AD can be terminated by inhibiting BACE1 (Boutajangout et al., 2011; Kwak et al., 2011; Yan and Vassar, 2014). The formation of BACE1 inhibitors, which is followed for many years, has still not been established as an effective treatment. However, constant improvement in this sphere has led to the formation of inhibitors that display widespread activity, from nano to micromolar. Consequently, evolving inhibitors for BACE1 have been an effective curative approach for AD drug discovery.

In this study, out of 1.4-M compounds in the database of Vitas-M Laboratory, 0.2-M complexes were selected, transferred, and arranged through phase (Dixon et al., 2006). Several groups of researchers have performed similar studies, such as for the discoveries of vaccines (Stokes et al., 2020), for *in silico* drug repositioning for AD (Galeana-Ascencio et al., 2023), and some other neurodegenerative diseases (Ishola et al., 2021). Others have performed similar approaches for *de novo* drug design (Wang et al., 2022). The Protein Data Bank (https://www.rcsb.org/) was used for retrieving the crystal structures of the BACE1 protein. One of the studies suggested the co-crystal ligands’ activity against BACE1, which was taken into consideration (Stamford and Strickland, 2013; Egbertson et al., 2015; Gupta et al., 2020). The pharmacophore model, which was receptor–ligand-based, was developed according to the inhibitor with the highest activity against BACE1. The Schrödinger phase tool acquired the pharmacophore hypothesis (Dixon et al., 2006).

Some previously reported BACE1 inhibitors, verubecestat (MK-8931) and its analog umibecestat (CNP-520), reached phase II/III clinical trials (Neumann et al., 2018; Thaisrivongs et al., 2018). However, these inhibitors were discontinued in February 2018 (Merck 2018.) and July 2019 (NIA, 2019), respectively, because they were associated with a decline in cognitive functions in participants.

In the current study, the two compounds S1 and S2 exhibited many hydrophobic and hydrogen bonding including hydrophobic interactions of S1; Gln>73, Gly>74, Leu>91, Asp>93, Trp>137, Trp>176, and Ser>290; hydrogen binding of Val>130, Tyr>132, Ile>179, and Gly>291; S2 hydrophobic interactions including Gly>74, Leu>91, and Asp>93; and hydrogen bonding of Gln>73, Val>130, Tyr>132, Trp>137, Trp>176, Ile>179, Ser>290, and Gly>291 (Figure 3). The previous study indicated that the AM-6494 inhibitor demonstrated active binding with BACE1, primarily involving interactions with specific amino acid residues including Lys>9, Gly>11, Gly>13, Tyr>14, Leu>30, Gly>34, Tyr>71, Thr>72, Phe>108, Trp>115, Ile>118, Val>170, Gly>230, Thr>231, Thr>232, Arg>235, Arg>307, and Ala>335. It is noteworthy that these interactions occur alongside the protonated Asp>32 and Asp>228 catalytic dyad (Ugbaja et al., 2021). The study also reported that 19 amino acid residues demonstrated interactions with the CNP-520 ligand, in which eight amino acids (Ser>35, Thr>72, Ile>110, Trp>115, Thr>231, Thr>232, Arg>235, and Ala>335) formed van der Waals (vdW) interactions (Ugbaja et al., 2021).

BACE1 harbors two aspartate amino acids (aa) within its extracellular protein domain (aa 93–96 and 289–292), both crucial for its protease function (Hussain et al., 1999). These residues are strategically located to facilitate the cleavage of APP at the β-site. In our study, both ligands S1 and S2 are responsible for hydrogen bonding with residues Asp>93, Gly>291, and Ser>290.

The protein-binding pocket and ligand sites were targeted for building the hypothesis. Virtual screening of pharmacophores has shown beneficial for hit identification and lead optimization in the initial phase of new drug development programs (Gautam et al., 2023). The main advantage of this approach is that virtually, millions of compounds can be screened for hit identification. Recently, virtual screening has been an obligatory part of drug research and development pipeline and an essential technique for discovering hits or chemical probes (Kumar and Zhang, 2015; Leung and Ma, 2015). Database screening from Vitas-M Laboratory library was executed virtually. At least four features must be matched to identify a complex as a hit. The final ranking of hits from the screening, contributing to the phase fitness score, was determined by vector arrangements, volume scores, and matching RMSD sites. The structures of the BACE1 protein were recovered from the Protein Data Bank. The literature was searched for the IC_50_ cut-offs of the co-crystal ligands. As evident, the ligand of crystal 66H showed maximum performance against the protease protein between the considered ligands. The identification of PDB, the arrangements, and the complementary IC_50_ values of other ligand studies are shown in Table 1. The compound STK081237 (chemical name: 6-hydrazinyl-N'-(naphthalen-1-yl)-N,N-diphenyl-1,3,5-triazine-2,4-diamine and empirical formula: C25H21N7) showed the align score of 0.31. Three compounds STK280616 (chemical name: ethyl4-phenyl-2-[(phenylcarbonyl) amino]thiophene-3-carboxylate and empirical formula: C20H17NO3S), compound STK057995 (chemical name: ethyl4-(3,4-dimethoxyphenyl)-2-[(pyridin-4-ylcarbonyl)amino]thiophene-3-carboxylate and empirical formula: C21H20N2O5S), and compound STK408850 (chemical name: ethyl4-(4-fluorophenyl)-2-[(3-methoxyphenyl)carbonyl]amino thiophene-3-carboxylate and empirical formula: C21H18FNO4S) obtained similar results of 0.453 align scores (Table 3). Recently, pharmacophore-based virtual screening and molecular docking studies of cyclin-dependent kinase inhibitors (CDKIs) have been reported (Shawky et al., 2021). Others studied applying pharmacophore modeling techniques to protease inhibitor development (Pautasso et al., 2014). A group of researchers recently reviewed the general aspects of AI and ML from the perspective of drug discovery in the CNS (Gautam et al., 2023). It is found that the co-crystal ligand 66H has the highest activity against BACE1 and can be potentially considered an inhibitor in drug development.

## 5 Conclusion

BACE1 is one of the most critical membrane-associated aspartate proteases that targets AD. Several inhibitors of ΒΑCE1 have been introduced, but effective therapies are still unavailable. Here, we attempted to find the most effective inhibitors against ΒΑCE1 for drug development against AD. We downloaded and prepared 200,000 compounds from the Vitas-M Laboratory database for virtual screening. We generated 10 conformers for each ligand to enhance the search in chemical space. It was found that among the studied ligands, the 66H crystal ligand exhibited the maximum performance against the protein. Our study provides a new perception of using 66H as anti ΒΑCE1 for drug development against AD.

## Data Availability

Publicly available datasets were analyzed in this study. These data can be found in the manuscript and are also available here: https://vitasmlab.biz.
