# Supplementary material for: Pharmacophore-based virtual screening of commercial databases against β-secretase 1 for drug development against Alzheimer’s disease
Source: Front Chem. 2024 Jul 9;12:1412349. doi: 10.3389/fchem.2024.1412349 (PMC11263123; doi:10.3389/fchem.2024.1412349)
Supplement: Supplementary file 1 [file DataSheet2.PDF]

Pharmacophore-based virtual screening of commercial databases against β-secretase 1 for the drug development against Alzheimer's disease

Supplementary tables

Table S1. Physiochemical Properties of 84 compounds

ID	MW	Vol	Dense	nHA	nHD	TPSA	Number of Rotation	Number of Het atoms	LogS	LogD	LogP
STK081237	419.19	438.241	0.957	7	4	98.45	4	7	-5.358	4.535	5.201
STK280616	351.09	358.319	0.98	4	1	55.4	7	5	-6.074	4.17	4.342
STK057995	412.11	404.192	1.02	7	1	86.75	9	8	-5.044	3.231	3.19
STK408850	399.09	390.472	1.022	5	1	64.63	8	7	-6.51	3.976	4.591
STK067256	358.04	338.573	1.057	5	1	68.29	7	7	-4.77	3.436	3.34
STK100429	455.03	423.282	1.075	4	1	58.11	6	8	-6.713	4.656	5.544
STK362117	327.09	343.276	0.953	4	1	63.24	3	4	-6.544	3.178	4.257
STK387431	396.08	384.259	1.031	7	1	98.54	8	8	-6.074	4.182	4.221
STK046443	369.06	318.423	1.159	6	2	82.33	4	10	-3.676	4.004	3.703
STK136267	289.03	261.791	1.104	5	1	71.25	3	7	-3.502	2.751	2.767
STK386021	317.03	291.528	1.087	5	1	67.34	5	7	-3.218	3.066	2.628
STK137950	299.05	284.956	1.049	4	1	58.11	3	6	-4.322	3.464	3.25
STK385466	366.97	315.378	1.164	4	1	58.11	3	8	-5.892	3.943	4.431
STK385674	363	326.606	1.111	4	1	58.11	3	7	-6.107	4.292	4.783
STK072483	329.04	311.395	1.057	4	1	58.11	3	6	-5.357	4.073	4.033
STK154114	345.03	320.186	1.078	5	1	67.34	4	7	-5.017	3.763	3.622
STK012551	329.04	311.395	1.057	4	1	58.11	3	6	-5.218	4.07	4.003
STK131655	289.03	261.791	1.104	5	1	71.25	3	7	-3.947	2.813	2.881
STK154089	329.06	311.042	1.058	5	1	67.34	4	7	-4.421	3.079	2.997
STK129297	350.99	306.739	1.144	5	1	67.34	5	8	-4.164	3.524	3.308
STK129615	313.07	302.252	1.036	4	1	58.11	3	6	-5.149	3.74	3.713
STK090091	416.91	339.733	1.227	4	1	58.11	3	9	-6.641	3.627	5.602
STK129509	272.04	249.424	1.091	6	1	84.14	3	7	-2.657	1.721	1.845
STK129571	299.05	284.956	1.049	4	1	58.11	3	6	-4.576	3.559	3.327
STK113693	444.03	378.586	1.173	6	1	76.18	4	8	-6.282	3.855	4.277
STK222598	322.09	331.141	0.973	3	2	41.13	5	4	-6.406	3.701	5.124
STK155936	332.08	331.108	1.003	6	1	79.62	4	6	-5.477	2.801	2.881

STK222602	366.04	335.214	1.092	3	2	41.13	5	4	-6.494	3.633	5.247
STK409019	288.04	268.09	1.074	4	1	58.36	3	6	-4.413	3.207	3.023
STK137196	339.1	339.566	0.999	5	1	67.34	5	6	-4.385	3.671	3.556
STK154090	329.06	311.042	1.058	5	1	67.34	4	7	-4.819	3.445	3.382
STK130675	348.98	309.31	1.128	4	1	58.11	3	7	-5.642	4.342	4.402
STK007472	331.04	308.824	1.072	5	1	67.34	5	7	-5.044	3.99	3.597
STK068025	325.11	323.386	1.005	5	2	69.19	3	6	-3.603	4.009	3.843
STK073398	329.04	311.395	1.057	4	1	58.11	3	6	-5.316	4.014	4.009
STK075179	313.07	302.252	1.036	4	1	58.11	3	6	-5.185	3.8	3.768
STK386018	309.09	313.48	0.986	4	1	58.11	3	5	-5.386	4.163	4.085
STK130489	298.06	291.255	1.023	3	1	45.22	3	5	-4.951	3.683	3.501
STK132568	311.07	304.974	1.02	5	1	67.34	5	6	-3.38	2.984	2.718
STK401920	304.01	277.233	1.097	4	1	58.36	3	6	-5.059	3.615	3.581
STK135071	329.04	311.395	1.057	4	1	58.11	3	6	-5.566	4.28	4.287
STK137123	348.98	309.31	1.128	4	1	58.11	3	7	-5.651	4.247	4.525
STK048780	372.99	315.468	1.182	4	1	58.11	3	6	-5.617	4.09	4.428
STK031760	392.93	313.383	1.254	4	1	58.11	3	7	-5.7	4.004	4.676
STK039660	372.99	315.468	1.182	4	1	58.11	3	6	-5.56	4.102	4.386
STK092252	363	326.606	1.111	4	1	58.11	3	7	-6.137	4.525	4.863
STK121703	336.1	348.437	0.965	3	2	41.13	5	4	-6.735	3.825	5.574
STK325732	295.08	296.184	0.996	4	1	58.11	3	5	-4.902	3.981	3.679
STK386010	297.06	287.678	1.033	5	1	71.25	4	6	-3.359	3.29	3.084
STK337539	270.05	262.022	1.031	4	1	58.36	3	5	-3.891	3.203	2.921
STK324799	330.05	315.123	1.047	4	1	54.45	4	6	-5.283	3.713	3.593
STK337540	280.07	285.187	0.982	3	1	45.22	3	4	-4.649	3.723	3.4
STK408865	318.02	294.529	1.08	4	1	58.36	3	6	-5.228	3.976	3.785
STK154061	313.07	302.252	1.036	4	1	58.11	3	6	-5.134	3.896	3.802
STK409037	312.07	308.551	1.011	3	1	45.22	3	5	-5.532	3.912	3.953
STK409038	316.05	297.323	1.063	3	1	45.22	3	6	-5.333	3.73	3.68
STK000255	388.98	324.258	1.2	5	1	67.34	4	7	-5.471	3.768	4.047
STK154110	343.08	328.338	1.045	5	1	67.34	5	7	-5.13	3.887	3.746

STK020405	325.09	322.27	1.009	5	1	67.34	4	6	-3.904	2.804	2.663
STK401922	347.99	315.61	1.103	3	1	45.22	3	6	-6.065	4.382	4.772
STK386029	339.07	322.504	1.051	6	1	76.57	3	7	-5.4	3.827	3.656
STK154130	355.12	354.14	1.003	4	1	58.11	4	6	-5.958	4.565	4.87
STK036626	347.99	315.61	1.103	3	1	45.22	3	6	-6.053	4.389	4.772
STK097228	329.08	316.401	1.04	6	1	80.48	5	7	-3.943	3.197	3.202
STK100419	298.06	291.255	1.023	3	1	45.22	3	5	-5.004	3.697	3.54
STK045387	325.09	322.27	1.009	5	1	67.34	5	6	-4.921	4.069	3.633
STK075062	450.95	330.485	1.365	6	1	76.57	3	8	-5.428	3.916	3.877
STK122203	312.07	308.551	1.011	3	1	45.22	3	5	-5.676	3.908	3.875
STK013762	343.04	311.276	1.102	6	1	76.57	3	8	-5.244	3.439	3.253
STK129898	325.09	322.27	1.009	5	1	67.34	4	6	-5.173	3.859	3.76
STK053591	402.96	324.492	1.242	6	1	76.57	3	8	-5.603	3.669	3.722
STK188417	345.03	320.186	1.078	5	1	67.34	4	7	-5.388	3.993	3.964
STK085958	359.07	337.128	1.065	6	1	76.57	5	8	-4.979	3.195	3.196
STK061013	345.06	326.12	1.058	5	1	67.34	6	7	-5.455	4.169	4.061
STK062148	344.04	310.897	1.107	7	1	101.25	4	9	-5.015	3.492	3.313
STK081664	340.06	322.125	1.056	7	1	101.25	4	8	-5.316	3.956	3.609
STK324798	358.05	343.781	1.042	4	1	54.45	4	6	-5.753	4.226	4.277
STK012081	403.96	324.113	1.246	7	1	101.25	4	9	-5.235	3.871	3.951
STK346841	349.06	339.735	1.027	5	1	68.02	4	6	-4.411	3.391	3.925
STK044786	326.05	304.829	1.07	7	1	101.25	4	8	-3.844	2.654	2.449
STK138023	301.03	282.738	1.065	4	1	58.11	3	6	-4.656	3.748	3.51
STK138208	320.98	280.653	1.144	4	1	58.11	3	7	-4.895	3.806	3.711
STK386019	311.07	304.974	1.02	5	1	67.34	4	6	-4.449	3.45	3.193
STK133249	343.08	328.338	1.045	5	1	67.34	5	7	-4.221	3.445	3.282

Table S2. Medicinal properties and Drug-Likeness of 84 compounds

ID	QED	Synth	Fsp3	MCE-18	Natural Product-likeness	Alarm_NMR	BMS	Chelating	PAINS	Lipinski	Pfizer	GSK	Golden Triangle
STK081237	0.298	3.039	0	24	-0.684	2	0	0	0	Accepted	Accepted	Rejected	Accepted
STK280616	0.669	1.797	0.1	16	-1.352	2	0	0	0	Accepted	Rejected	Rejected	Accepted

STK057995	0.584	2.151	0.19	18	-1.254	3	0	0	0	Accepted	Accepted	Rejected	Accepted
STK408850	0.593	1.982	0.143	18	-1.688	3	0	0	0	Accepted	Rejected	Rejected	Accepted
STK067256	0.691	2.226	0.118	16	-2.003	2	0	0	0	Accepted	Rejected	Accepted	Accepted
STK100429	0.377	2.676	0.045	21	-1.921	4	0	0	0	Accepted	Rejected	Rejected	Accepted
STK362117	0.61	1.725	0	42	-0.643	1	0	0	1	Accepted	Rejected	Rejected	Accepted
STK387431	0.368	2.041	0.1	18	-1.752	3	0	0	0	Accepted	Accepted	Rejected	Accepted
STK046443	0.742	3.432	0.133	18	-1.414	1	0	0	0	Accepted	Accepted	Accepted	Accepted
STK136267	0.788	2.855	0	15	-2.022	3	0	0	0	Accepted	Accepted	Accepted	Accepted
STK386021	0.804	2.775	0.071	14	-2.031	4	0	0	0	Accepted	Accepted	Accepted	Accepted
STK137950	0.79	2.513	0	15	-1.805	3	0	0	0	Accepted	Rejected	Accepted	Accepted
STK385466	0.732	2.627	0	17	-2.1	3	0	0	0	Accepted	Rejected	Rejected	Accepted
STK385674	0.728	2.607	0.062	17	-1.881	3	0	0	0	Accepted	Rejected	Rejected	Accepted
STK072483	0.776	2.528	0.062	16	-1.826	3	0	0	0	Accepted	Rejected	Rejected	Accepted
STK154114	0.789	2.574	0.062	16	-1.653	4	0	0	0	Accepted	Rejected	Accepted	Accepted
STK012551	0.776	2.591	0.062	16	-1.944	3	0	0	0	Accepted	Rejected	Rejected	Accepted
STK131655	0.788	2.81	0	15	-2.057	3	0	0	0	Accepted	Accepted	Accepted	Accepted
STK154089	0.803	2.582	0.062	16	-1.662	4	0	0	0	Accepted	Accepted	Accepted	Accepted
STK129297	0.782	2.805	0.071	15	-2.17	4	0	0	0	Accepted	Rejected	Accepted	Accepted
STK129615	0.789	2.541	0.062	16	-1.908	3	0	0	0	Accepted	Rejected	Accepted	Accepted
STK090091	0.597	2.725	0	18	-1.907	3	0	0	0	Accepted	Rejected	Rejected	Accepted
STK129509	0.771	2.998	0	14	-1.882	3	0	0	0	Accepted	Accepted	Accepted	Accepted
STK129571	0.79	2.47	0	15	-1.86	3	0	0	0	Accepted	Rejected	Accepted	Accepted
STK113693	0.505	3.039	0.211	23	-2.248	4	0	0	0	Accepted	Accepted	Rejected	Accepted
STK222598	0.681	1.508	0	15	-1.452	2	0	0	0	Accepted	Rejected	Rejected	Accepted
STK155936	0.746	1.925	0	42	-1.743	1	0	0	0	Accepted	Accepted	Accepted	Accepted
STK222602	0.644	1.556	0	15	-1.332	3	0	0	0	Accepted	Rejected	Rejected	Accepted
STK409019	0.787	2.762	0	15	-2.186	3	0	0	0	Accepted	Rejected	Accepted	Accepted
STK137196	0.792	2.67	0.167	16	-1.447	4	0	0	0	Accepted	Rejected	Accepted	Accepted
STK154090	0.803	2.573	0.062	16	-1.831	4	0	0	0	Accepted	Rejected	Accepted	Accepted
STK130675	0.754	2.577	0	16	-1.822	3	0	0	0	Accepted	Rejected	Rejected	Accepted
STK007472	0.797	2.731	0.133	15	-2.061	4	0	0	0	Accepted	Rejected	Accepted	Accepted

STK068025	0.763	3.039	0.118	15	-0.954	1	0	0	0	Accepted	Rejected	Accepted	Accepted
STK073398	0.776	2.591	0.062	16	-1.767	3	0	0	0	Accepted	Rejected	Rejected	Accepted
STK075179	0.789	2.499	0.062	16	-1.916	3	0	0	0	Accepted	Rejected	Accepted	Accepted
STK386018	0.787	2.478	0.118	16	-1.509	3	0	0	0	Accepted	Rejected	Rejected	Accepted
STK130489	0.771	2.424	0	15	-1.983	3	0	0	0	Accepted	Rejected	Accepted	Accepted
STK132568	0.805	2.477	0.062	14	-1.543	4	0	0	0	Accepted	Accepted	Accepted	Accepted
STK401920	0.784	2.755	0	15	-2.074	3	0	0	0	Accepted	Rejected	Accepted	Accepted
STK135071	0.776	2.493	0.062	16	-1.814	3	0	0	0	Accepted	Rejected	Rejected	Accepted
STK137123	0.754	2.509	0	16	-1.636	3	0	0	0	Accepted	Rejected	Rejected	Accepted
STK048780	0.739	2.552	0.062	16	-1.691	4	0	0	0	Accepted	Rejected	Rejected	Accepted
STK031760	0.704	2.568	0	16	-1.818	4	0	0	0	Accepted	Rejected	Rejected	Accepted
STK039660	0.739	2.616	0.062	16	-1.832	4	0	0	0	Accepted	Rejected	Rejected	Accepted
STK092252	0.728	2.644	0.062	17	-1.863	3	0	0	0	Accepted	Rejected	Rejected	Accepted
STK121703	0.647	1.584	0.05	16	-1.527	2	0	0	0	Accepted	Rejected	Rejected	Accepted
STK325732	0.789	2.438	0.062	15	-1.662	3	0	0	0	Accepted	Rejected	Accepted	Accepted
STK386010	0.808	2.989	0	14	-1.385	3	0	0	0	Accepted	Rejected	Accepted	Accepted
STK337539	0.778	2.709	0	14	-1.892	3	0	0	0	Accepted	Accepted	Accepted	Accepted
STK324799	0.793	2.671	0.125	16	-1.668	4	0	0	0	Accepted	Rejected	Accepted	Accepted
STK337540	0.767	2.36	0	14	-1.654	3	0	0	0	Accepted	Rejected	Accepted	Accepted
STK408865	0.775	2.81	0.067	16	-1.843	3	0	0	0	Accepted	Rejected	Accepted	Accepted
STK154061	0.789	2.626	0.062	16	-2.183	3	0	0	0	Accepted	Rejected	Accepted	Accepted
STK409037	0.765	2.454	0.059	16	-2.034	3	0	0	0	Accepted	Rejected	Accepted	Accepted
STK409038	0.768	2.545	0	16	-2.132	3	0	0	0	Accepted	Rejected	Accepted	Accepted
STK000255	0.743	2.526	0.062	16	-1.508	5	0	0	0	Accepted	Rejected	Rejected	Accepted
STK154110	0.789	2.597	0.118	16	-1.999	4	0	0	0	Accepted	Rejected	Accepted	Accepted
STK020405	0.804	2.602	0.118	16	-1.402	4	0	0	0	Accepted	Accepted	Accepted	Accepted
STK401922	0.71	2.464	0	16	-1.754	3	0	0	0	Accepted	Rejected	Rejected	Accepted
STK386029	0.779	2.68	0.118	42.105	-1.308	3	0	0	0	Accepted	Accepted	Accepted	Accepted
STK154130	0.744	2.659	0.211	19	-1.861	3	0	0	0	Accepted	Rejected	Rejected	Accepted
STK036626	0.71	2.53	0	16	-2.002	3	0	0	0	Accepted	Rejected	Rejected	Accepted
STK097228	0.798	2.918	0.188	16	-1.793	4	0	0	0	Accepted	Accepted	Accepted	Accepted

STK100419	0.771	2.414	0	15	-2.025	3	0	0	0	Accepted	Rejected	Accepted	Accepted
STK045387	0.8	2.447	0.118	15	-1.628	4	0	0	0	Accepted	Rejected	Accepted	Accepted
STK075062	0.608	2.902	0.062	42.353	-1.265	4	0	0	0	Accepted	Accepted	Rejected	Accepted
STK122203	0.765	2.523	0.059	16	-2.074	3	0	0	0	Accepted	Rejected	Accepted	Accepted
STK013762	0.776	2.761	0.062	42.353	-1.555	3	0	0	0	Accepted	Accepted	Accepted	Accepted
STK129898	0.804	2.51	0.118	16	-1.642	4	0	0	0	Accepted	Rejected	Accepted	Accepted
STK053591	0.711	2.812	0.062	42.353	-1.266	4	0	0	0	Accepted	Accepted	Rejected	Accepted
STK188417	0.789	2.528	0.062	16	-1.763	4	0	0	0	Accepted	Rejected	Accepted	Accepted
STK085958	0.777	2.599	0.118	17	-1.619	4	0	0	0	Accepted	Accepted	Accepted	Accepted
STK061013	0.766	2.743	0.188	15	-1.94	4	0	0	0	Accepted	Rejected	Rejected	Accepted
STK062148	0.583	2.615	0	17	-2.066	4	0	0	0	Accepted	Accepted	Accepted	Accepted
STK081664	0.584	2.595	0.062	17	-1.856	4	0	0	0	Accepted	Accepted	Accepted	Accepted
STK324798	0.751	2.433	0.111	17	-1.445	4	0	0	0	Accepted	Rejected	Rejected	Accepted
STK012081	0.532	2.664	0	17	-1.86	5	0	0	0	Accepted	Accepted	Rejected	Accepted
STK346841	0.577	1.984	0	20	-1.97	0	0	0	0	Accepted	Rejected	Accepted	Accepted
STK044786	0.59	2.741	0	16	-2.065	4	0	0	0	Accepted	Accepted	Accepted	Accepted
STK138023	0.79	2.745	0.071	15	-2.238	3	0	0	0	Accepted	Rejected	Accepted	Accepted
STK138208	0.784	2.762	0	15	-2.378	3	0	0	0	Accepted	Rejected	Accepted	Accepted
STK386019	0.808	2.483	0.062	15	-1.403	4	0	0	0	Accepted	Rejected	Accepted	Accepted
STK133249	0.791	2.613	0.118	16	-2.025	4	0	0	0	Accepted	Rejected	Accepted	Accepted

Table S3. Absorption and Distribution properties of 84 compounds

ID	Pgp-Inhibitor	Pgp-Substrate	HIA	F(20%)	F(30%)	Caco-2	MDCK	BBB	PPB	VDss	Fu
STK081237	0.02	0.014	0.748	0.924	0.003	-4.565	4.96E-05	0.209	91.51%	3.085	4.46%
STK280616	0.77	0	0.003	0.011	0.879	-4.656	1.48E-05	0.073	98.73%	0.931	0.63%
STK057995	0.973	0	0.004	0.007	0.032	-4.874	2.36E-05	0.196	96.09%	0.834	2.55%
STK408850	0.996	0	0.003	0.005	0.05	-4.828	1.93E-05	0.071	99.43%	0.704	0.79%
STK067256	0.071	0	0.008	0.015	0.294	-4.325	5.98E-05	0.064	96.45%	0.788	2.81%
STK100429	0.163	0.002	0.062	0.001	0.019	-4.738	1.23E-05	0.27	100.45%	0.728	0.58%
STK362117	0.996	0.002	0.013	0.006	0.997	-4.922	1.39E-05	0.068	101.07%	0.96	0.70%
STK387431	0.874	0	0.004	0.004	0.012	-4.636	0.000109	0.024	99.35%	0.685	0.72%

STK046443	0.011	0.03	0.009	0.001	0	-3.918	3.11E-05	0.408	95.58%	3.089	5.40%
STK136267	0.007	0.002	0.012	0.001	0.01	-4.731	2.10E-05	0.694	94.84%	1.512	4.31%
STK386021	0.002	0	0.816	0.027	0.192	-4.568	2.32E-05	0.396	93.97%	0.66	1.60%
STK137950	0.006	0.001	0.066	0.001	0.005	-4.569	2.95E-05	0.761	94.74%	0.59	2.86%
STK385466	0.024	0.002	0.005	0.001	0.007	-4.66	1.35E-05	0.54	99.70%	0.682	1.19%
STK385674	0.034	0.003	0.005	0.002	0.007	-4.694	1.41E-05	0.544	99.55%	0.764	1.41%
STK072483	0.013	0.003	0.007	0.002	0.002	-4.624	2.13E-05	0.731	97.75%	0.703	1.79%
STK154114	0.007	0.004	0.031	0.001	0.011	-4.622	1.75E-05	0.682	98.03%	0.661	1.66%
STK012551	0.012	0.004	0.009	0.002	0.003	-4.63	2.20E-05	0.749	97.77%	0.697	1.84%
STK131655	0.025	0.003	0.013	0.001	0.005	-4.742	1.98E-05	0.716	95.13%	1.764	4.54%
STK154089	0.009	0.002	0.009	0.001	0.012	-4.597	2.96E-05	0.725	95.31%	0.655	2.87%
STK129297	0.003	0	0.109	0.004	0.164	-4.605	1.70E-05	0.257	98.39%	0.701	1.15%
STK129615	0.015	0.003	0.007	0.001	0.002	-4.581	2.70E-05	0.775	96.29%	0.612	2.79%
STK090091	0.038	0.001	0.004	0.002	0.785	-4.8	9.16E-06	0.145	101.07%	1.51	1.65%
STK129509	0.001	0.002	0.041	0.004	0.005	-4.636	1.94E-05	0.825	67.28%	1.018	41.24%
STK129571	0.014	0.002	0.055	0.001	0.003	-4.596	2.29E-05	0.795	95.12%	0.605	3.06%
STK113693	0.139	0.001	0.611	0.002	0.002	-5.019	1.42E-05	0.743	97.50%	0.882	2.95%
STK222598	0.148	0.167	0.403	0.865	0.713	-4.821	9.50E-06	0.098	99.07%	1.232	0.69%
STK155936	0.298	0.002	0.066	0.022	0.086	-4.815	1.14E-05	0.22	95.53%	0.707	4.53%
STK222602	0.822	0.003	0.012	0.026	0.031	-4.819	9.69E-06	0.207	98.62%	1.239	0.99%
STK409019	0.909	0.001	0.005	0.001	0.081	-4.787	1.95E-05	0.512	95.68%	2.075	2.83%
STK137196	0.045	0.041	0.005	0.001	0.001	-4.767	2.43E-05	0.689	96.46%	0.517	2.16%
STK154090	0.021	0.002	0.013	0.001	0.009	-4.614	2.03E-05	0.751	96.50%	0.748	2.48%
STK130675	0.013	0.001	0.012	0.002	0.047	-4.685	1.63E-05	0.446	98.92%	0.8	1.33%
STK007472	0.016	0	0.187	0.006	0.501	-4.463	3.06E-05	0.303	97.56%	0.647	1.32%
STK068025	0.01	0.211	0.013	0.003	0.001	-3.907	4.90E-05	0.102	93.93%	1.79	7.47%
STK073398	0.009	0.002	0.01	0.002	0.003	-4.669	2.74E-05	0.748	97.70%	0.707	1.55%
STK075179	0.044	0.008	0.007	0.001	0.002	-4.604	2.32E-05	0.79	96.52%	0.618	2.96%
STK386018	0.109	0.062	0.005	0.002	0.001	-4.648	2.59E-05	0.781	96.46%	0.655	3.07%
STK130489	0.82	0	0.004	0.001	0.011	-4.782	1.69E-05	0.678	96.19%	0.609	1.85%
STK132568	0.003	0.001	0.106	0.003	0.001	-4.726	2.30E-05	0.621	91.16%	0.623	4.12%

STK401920	0.745	0.001	0.004	0.001	0.005	-4.851	1.70E-05	0.271	97.82%	1.998	2.06%
STK135071	0.018	0.008	0.009	0.001	0.001	-4.638	1.76E-05	0.627	98.12%	0.641	1.84%
STK137123	0.016	0.002	0.021	0.002	0.004	-4.652	1.20E-05	0.405	98.95%	0.585	1.33%
STK048780	0.193	0.001	0.006	0.001	0.001	-4.617	1.91E-05	0.781	97.43%	0.763	2.64%
STK031760	0.102	0.001	0.007	0.001	0.001	-4.632	1.41E-05	0.644	98.35%	0.754	1.66%
STK039660	0.15	0.002	0.006	0.001	0.001	-4.626	1.89E-05	0.809	97.33%	0.619	2.92%
STK092252	0.018	0.002	0.005	0.002	0.017	-4.747	1.72E-05	0.563	99.33%	0.74	1.46%
STK121703	0.717	0.088	0.022	0.383	0.378	-4.819	1.03E-05	0.092	99.32%	1.704	0.65%
STK325732	0.006	0.009	0.02	0.002	0.001	-4.606	2.57E-05	0.79	95.32%	0.669	3.63%
STK386010	0.187	0.007	0.105	0.002	0.001	-4.775	1.90E-05	0.428	98.01%	2.352	2.15%
STK337539	0.49	0.001	0.007	0.012	0.007	-4.831	2.08E-05	0.549	94.12%	1.985	4.31%
STK324799	0.041	0	0.004	0.003	0.029	-4.782	1.97E-05	0.321	97.50%	0.55	1.18%
STK337540	0.275	0.001	0.006	0.051	0.003	-4.776	1.75E-05	0.667	94.23%	0.673	3.18%
STK408865	0.057	0.002	0.005	0.002	0.002	-4.951	1.82E-05	0.214	98.52%	1.523	1.62%
STK154061	0.036	0.012	0.01	0.001	0.002	-4.619	2.42E-05	0.85	96.46%	0.643	3.19%
STK409037	0.907	0.001	0.003	0.001	0.006	-4.768	1.69E-05	0.685	97.31%	0.632	1.45%
STK409038	0.989	0	0.003	0.001	0.01	-4.744	1.69E-05	0.71	97.95%	0.578	0.95%
STK000255	0.54	0.001	0.007	0.001	0.003	-4.626	1.60E-05	0.783	97.38%	0.675	3.55%
STK154110	0.037	0.001	0.007	0.001	0.037	-4.606	2.07E-05	0.591	97.83%	0.587	1.62%
STK020405	0.016	0.001	0.004	0.001	0.001	-4.771	2.34E-05	0.947	86.42%	0.882	11.09%
STK401922	0.583	0	0.003	0.002	0.003	-4.849	1.01E-05	0.238	99.42%	0.594	0.88%
STK386029	0.029	0.006	0.004	0.001	0.093	-4.66	1.94E-05	0.744	97.54%	0.701	2.39%
STK154130	0.901	0.004	0.005	0.001	0.003	-4.792	2.58E-05	0.561	98.47%	0.851	1.65%
STK036626	0.629	0	0.003	0.002	0.003	-4.86	1.00E-05	0.197	99.72%	0.434	0.82%
STK097228	0.004	0.018	0.004	0.001	0.002	-4.819	1.88E-05	0.51	95.80%	1.093	6.35%
STK100419	0.815	0	0.004	0.001	0.011	-4.783	1.69E-05	0.679	96.18%	0.612	1.85%
STK045387	0.023	0.001	0.011	0.002	0.028	-4.607	2.13E-05	0.568	97.07%	0.548	2.14%
STK075062	0.012	0.001	0.005	0.001	0.045	-4.624	1.96E-05	0.628	98.31%	0.58	1.61%
STK122203	0.659	0.001	0.003	0.001	0.004	-4.816	1.79E-05	0.679	97.17%	0.558	1.45%
STK013762	0.016	0.001	0.004	0.001	0.042	-4.659	2.29E-05	0.746	97.75%	0.713	2.45%
STK129898	0.088	0.018	0.008	0.001	0.003	-4.687	2.04E-05	0.794	96.48%	0.677	3.08%

STK053591	0.793	0	0.004	0.002	0.023	-4.635	1.65E-05	0.757	98.30%	0.666	2.65%
STK188417	0.018	0.004	0.039	0.001	0.004	-4.676	1.56E-05	0.593	98.28%	0.688	1.72%
STK085958	0.806	0.006	0.005	0.002	0.015	-4.641	1.63E-05	0.662	95.84%	0.759	3.22%
STK061013	0.018	0.001	0.315	0.018	0.641	-4.474	3.61E-05	0.313	98.11%	0.7	1.24%
STK062148	0.02	0.002	0.033	0.001	0.001	-4.605	0.000171	0.452	96.87%	0.616	1.94%
STK081664	0.004	0.007	0.019	0.001	0	-4.589	0.00015	0.538	96.74%	0.745	2.61%
STK324798	0.119	0.001	0.003	0.002	0.002	-4.88	1.44E-05	0.31	98.99%	0.56	0.84%
STK012081	0.108	0.001	0.007	0.001	0	-4.577	0.000103	0.538	97.74%	0.662	1.72%
STK346841	0.987	0.002	0.009	0.033	0.372	-4.912	1.39E-05	0.402	98.40%	2.599	1.56%
STK044786	0.031	0	0.007	0.001	0	-4.651	7.05E-05	0.722	77.32%	0.731	19.01%
STK138023	0.001	0.001	0.12	0.012	0.039	-4.49	4.50E-05	0.488	95.86%	0.655	2.85%
STK138208	0.004	0	0.04	0.008	0.148	-4.471	3.30E-05	0.275	97.85%	0.699	1.73%
STK386019	0.006	0.003	0.083	0.002	0.029	-4.625	2.67E-05	0.81	92.56%	0.513	4.59%
STK133249	0.045	0.003	0.005	0.001	0.002	-4.674	2.26E-05	0.619	96.74%	0.607	2.13%

Table S4. Metabolism and Excretion properties of 84 compounds

	**Metabolism**										**Excretion**	
**ID**	CYP1A2-Inhibitor	CYP1A2-Substrate	CYP2C19-Inhibitor	CYP2C19-Substrate	CYP2C9-Inhibitor	CYP2C9-Substrate	CYP2D6-In.	CYP2D6-Substrate	CYP3A4-Inhibitor	CYP3A4-Substrate	CL	T12
STK081237	0.816	0.86	0.845	0.048	0.569	0.002	0.029	0	0.843	0.949	5.071	0.214
STK280616	0.931	0.15	0.944	0.072	0.923	0.743	0.224	0.314	0.32	0.194	4.779	0.058
STK057995	0.911	0.63	0.951	0.16	0.958	0.784	0.867	0.648	0.96	0.655	9.075	0.23
STK408850	0.86	0.569	0.906	0.072	0.91	0.864	0.31	0.876	0.555	0.23	6.405	0.03
STK067256	0.977	0.158	0.953	0.079	0.953	0.502	0.934	0.351	0.95	0.36	3.131	0.064
STK100429	0.868	0.184	0.939	0.059	0.974	0.833	0.659	0.242	0.672	0.373	0.933	0.015
STK362117	0.743	0.087	0.598	0.059	0.619	0.286	0.127	0.177	0.456	0.153	1.085	0.077
STK387431	0.855	0.108	0.914	0.067	0.932	0.754	0.281	0.363	0.557	0.151	3.939	0.06
STK046443	0.706	0.948	0.947	0.049	0.626	0.006	0.028	0.001	0.797	0.918	6.883	0.659
STK136267	0.979	0.122	0.966	0.06	0.933	0.802	0.441	0.102	0.355	0.187	0.977	0.094
STK386021	0.981	0.222	0.97	0.066	0.946	0.906	0.509	0.161	0.574	0.397	0.992	0.153
STK137950	0.97	0.118	0.961	0.062	0.936	0.817	0.464	0.097	0.261	0.199	0.573	0.075
STK385466	0.949	0.184	0.953	0.06	0.939	0.605	0.693	0.131	0.339	0.219	0.787	0.026

STK385674	0.925	0.408	0.955	0.063	0.951	0.661	0.709	0.174	0.531	0.336	0.836	0.039
STK072483	0.941	0.336	0.961	0.064	0.947	0.764	0.661	0.173	0.574	0.354	0.697	0.056
STK154114	0.969	0.8	0.969	0.068	0.943	0.821	0.746	0.151	0.765	0.289	1.124	0.124
STK012551	0.957	0.526	0.967	0.064	0.943	0.746	0.69	0.143	0.806	0.316	0.803	0.079
STK131655	0.978	0.128	0.964	0.057	0.927	0.728	0.516	0.109	0.33	0.2	1.071	0.088
STK154089	0.966	0.648	0.968	0.079	0.938	0.883	0.653	0.21	0.708	0.273	1.216	0.079
STK129297	0.978	0.476	0.973	0.067	0.955	0.872	0.751	0.225	0.742	0.53	0.842	0.084
STK129615	0.937	0.252	0.961	0.064	0.943	0.77	0.635	0.196	0.553	0.275	0.596	0.057
STK090091	0.955	0.212	0.947	0.061	0.949	0.616	0.702	0.13	0.417	0.3	1.182	0.024
STK129509	0.983	0.139	0.964	0.055	0.944	0.357	0.39	0.067	0.677	0.233	0.972	0.445
STK129571	0.965	0.126	0.961	0.06	0.93	0.709	0.513	0.103	0.262	0.212	0.629	0.077
STK113693	0.925	0.876	0.948	0.216	0.971	0.723	0.645	0.679	0.935	0.892	4.261	0.078
STK222598	0.775	0.182	0.91	0.067	0.625	0.718	0.647	0.587	0.341	0.361	3.458	0.311
STK155936	0.586	0.088	0.776	0.089	0.87	0.847	0.044	0.345	0.095	0.4	0.528	0.227
STK222602	0.762	0.148	0.902	0.068	0.637	0.725	0.636	0.632	0.33	0.289	1.76	0.316
STK409019	0.989	0.149	0.959	0.055	0.898	0.253	0.122	0.281	0.077	0.25	3.194	0.145
STK137196	0.937	0.779	0.966	0.089	0.945	0.814	0.081	0.187	0.843	0.518	1.346	0.323
STK154090	0.966	0.616	0.967	0.066	0.942	0.874	0.722	0.297	0.812	0.22	0.985	0.085
STK130675	0.965	0.18	0.966	0.06	0.954	0.733	0.695	0.104	0.465	0.31	1.015	0.068
STK007472	0.957	0.273	0.962	0.062	0.942	0.868	0.757	0.344	0.59	0.306	0.758	0.061
STK068025	0.785	0.939	0.936	0.051	0.399	0.009	0.039	0.001	0.697	0.927	6.642	0.823
STK073398	0.959	0.477	0.968	0.066	0.945	0.844	0.706	0.162	0.687	0.346	0.782	0.037
STK075179	0.919	0.234	0.953	0.061	0.934	0.66	0.63	0.222	0.428	0.301	0.696	0.058
STK386018	0.854	0.633	0.953	0.064	0.938	0.705	0.57	0.336	0.566	0.478	0.74	0.093
STK130489	0.979	0.144	0.941	0.057	0.913	0.364	0.107	0.161	0.069	0.279	1.873	0.118
STK132568	0.969	0.104	0.96	0.065	0.946	0.922	0.044	0.098	0.174	0.329	1.145	0.328
STK401920	0.991	0.19	0.967	0.057	0.925	0.223	0.278	0.189	0.085	0.331	2.553	0.178
STK135071	0.926	0.351	0.959	0.062	0.946	0.634	0.712	0.149	0.431	0.422	0.747	0.072
STK137123	0.957	0.201	0.963	0.06	0.951	0.522	0.731	0.09	0.289	0.352	0.769	0.051
STK048780	0.912	0.185	0.95	0.062	0.946	0.654	0.652	0.17	0.454	0.319	0.76	0.065
STK031760	0.947	0.174	0.954	0.06	0.949	0.57	0.704	0.1	0.29	0.277	0.774	0.049

STK039660	0.946	0.283	0.961	0.062	0.943	0.63	0.693	0.139	0.701	0.28	0.818	0.091
STK092252	0.95	0.449	0.96	0.063	0.955	0.759	0.754	0.185	0.681	0.393	0.995	0.043
STK121703	0.587	0.22	0.812	0.075	0.621	0.824	0.478	0.843	0.354	0.619	3.61	0.213
STK325732	0.95	0.248	0.961	0.063	0.937	0.756	0.408	0.128	0.434	0.304	0.668	0.116
STK386010	0.979	0.108	0.966	0.063	0.941	0.835	0.074	0.235	0.065	0.259	1.366	0.217
STK337539	0.992	0.139	0.96	0.055	0.885	0.147	0.035	0.163	0.045	0.251	3.305	0.37
STK324799	0.975	0.904	0.957	0.073	0.941	0.772	0.77	0.782	0.896	0.701	3.09	0.113
STK337540	0.986	0.135	0.943	0.058	0.894	0.291	0.035	0.107	0.041	0.275	1.948	0.272
STK408865	0.982	0.581	0.963	0.059	0.941	0.119	0.177	0.12	0.302	0.437	3.892	0.224
STK154061	0.96	0.498	0.965	0.062	0.927	0.665	0.621	0.148	0.82	0.229	0.837	0.112
STK409037	0.955	0.258	0.919	0.063	0.894	0.372	0.207	0.468	0.212	0.528	1.892	0.089
STK409038	0.977	0.166	0.934	0.056	0.88	0.346	0.244	0.266	0.124	0.232	2.058	0.086
STK000255	0.922	0.422	0.955	0.064	0.949	0.855	0.697	0.322	0.562	0.281	0.907	0.067
STK154110	0.943	0.215	0.966	0.064	0.949	0.876	0.658	0.216	0.551	0.201	0.772	0.063
STK020405	0.964	0.797	0.939	0.109	0.923	0.719	0.536	0.521	0.942	0.611	5.141	0.475
STK401922	0.98	0.197	0.935	0.057	0.862	0.292	0.508	0.156	0.109	0.517	1.6	0.078
STK386029	0.951	0.174	0.968	0.061	0.938	0.703	0.852	0.234	0.919	0.279	2.416	0.129
STK154130	0.911	0.345	0.953	0.068	0.96	0.87	0.767	0.16	0.647	0.433	0.897	0.029
STK036626	0.984	0.207	0.952	0.056	0.915	0.317	0.484	0.169	0.117	0.339	1.794	0.1
STK097228	0.959	0.723	0.966	0.08	0.948	0.787	0.085	0.231	0.78	0.498	2.355	0.299
STK100419	0.979	0.144	0.939	0.057	0.912	0.37	0.104	0.162	0.066	0.271	1.858	0.116
STK045387	0.905	0.143	0.957	0.063	0.945	0.867	0.485	0.148	0.272	0.238	0.726	0.097
STK075062	0.975	0.164	0.965	0.062	0.938	0.702	0.832	0.179	0.942	0.207	1.929	0.082
STK122203	0.965	0.347	0.957	0.064	0.934	0.602	0.206	0.521	0.32	0.434	2.146	0.09
STK013762	0.973	0.148	0.974	0.062	0.94	0.802	0.823	0.16	0.932	0.188	3.054	0.088
STK129898	0.939	0.863	0.963	0.066	0.941	0.862	0.646	0.507	0.845	0.375	1.043	0.116
STK053591	0.971	0.149	0.974	0.062	0.946	0.701	0.832	0.153	0.933	0.166	1.39	0.101
STK188417	0.962	0.726	0.97	0.064	0.947	0.833	0.78	0.212	0.759	0.318	1.042	0.089
STK085958	0.951	0.823	0.959	0.078	0.924	0.817	0.529	0.303	0.813	0.292	2.795	0.192
STK061013	0.943	0.3	0.959	0.062	0.945	0.844	0.778	0.429	0.639	0.29	0.85	0.049
STK062148	0.894	0.081	0.937	0.054	0.888	0.822	0.611	0.146	0.37	0.179	0.588	0.053

STK081664	0.811	0.109	0.94	0.058	0.916	0.84	0.518	0.194	0.495	0.248	0.607	0.078
STK324798	0.933	0.826	0.929	0.066	0.948	0.635	0.599	0.462	0.54	0.756	3.666	0.098
STK012081	0.885	0.082	0.934	0.056	0.924	0.808	0.625	0.124	0.384	0.178	0.661	0.057
STK346841	0.961	0.14	0.925	0.062	0.824	0.615	0.819	0.657	0.923	0.198	3.476	0.158
STK044786	0.964	0.109	0.897	0.065	0.843	0.678	0.177	0.262	0.793	0.294	1.512	0.36
STK138023	0.971	0.541	0.963	0.064	0.939	0.77	0.702	0.286	0.754	0.322	0.912	0.054
STK138208	0.98	0.32	0.965	0.062	0.944	0.702	0.774	0.137	0.617	0.288	0.894	0.042
STK386019	0.975	0.689	0.967	0.076	0.93	0.874	0.515	0.133	0.601	0.308	1.544	0.141
STK133249	0.946	0.515	0.961	0.066	0.949	0.861	0.364	0.301	0.729	0.381	1.005	0.116

Table S5. Toxicology parameters of 84 compounds

ID	hERG Blockers	Human hepatotoxicity	Drug-induced liver injury	AMES Toxicity	Rat Oral Acute Toxicity	Maximum recommended daily dose	Skin Sensitization	Carcinogenicity	Eye Corrosion	Eye Irritation	Respiratory
STK081237	0.837	0.998	0.995	0.058	0.054	0.896	0.868	0.832	0.003	0.028	0.695
STK280616	0.241	0.051	0.974	0.03	0.015	0.018	0.601	0.351	0.003	0.574	0.24
STK057995	0.646	0.033	0.974	0.057	0.046	0.028	0.085	0.124	0.003	0.021	0.445
STK408850	0.432	0.155	0.949	0.058	0.046	0.134	0.136	0.286	0.003	0.139	0.143
STK067256	0.451	0.067	0.983	0.021	0.036	0.019	0.348	0.041	0.003	0.17	0.97
STK100429	0.129	0.981	0.992	0.915	0.079	0.592	0.062	0.802	0.003	0.207	0.462
STK362117	0.362	0.134	0.957	0.735	0.163	0.859	0.271	0.834	0.003	0.905	0.044
STK387431	0.635	0.151	0.957	0.91	0.032	0.064	0.794	0.661	0.003	0.522	0.506
STK046443	0.954	0.991	0.951	0.03	0.884	0.965	0.292	0.603	0.003	0.026	0.954
STK136267	0.042	0.983	0.984	0.826	0.899	0.032	0.046	0.837	0.004	0.659	0.962
STK386021	0.005	0.964	0.987	0.954	0.119	0.029	0.181	0.922	0.003	0.22	0.626
STK137950	0.016	0.983	0.987	0.566	0.141	0.035	0.079	0.87	0.004	0.831	0.87
STK385466	0.091	0.979	0.987	0.155	0.42	0.534	0.058	0.707	0.003	0.171	0.505
STK385674	0.078	0.965	0.987	0.155	0.369	0.099	0.061	0.694	0.003	0.345	0.529
STK072483	0.033	0.966	0.988	0.246	0.388	0.032	0.073	0.8	0.004	0.783	0.73
STK154114	0.045	0.962	0.986	0.357	0.07	0.042	0.071	0.78	0.003	0.332	0.576
STK012551	0.027	0.965	0.987	0.183	0.232	0.038	0.08	0.754	0.004	0.74	0.789
STK131655	0.064	0.978	0.983	0.703	0.857	0.13	0.046	0.852	0.004	0.341	0.954
STK154089	0.023	0.984	0.986	0.803	0.214	0.039	0.052	0.909	0.004	0.404	0.735
STK129297	0.009	0.963	0.987	0.928	0.348	0.04	0.113	0.922	0.003	0.075	0.44

STK129615	0.021	0.983	0.987	0.657	0.425	0.033	0.061	0.9	0.004	0.732	0.791
STK090091	0.154	0.952	0.988	0.024	0.139	0.495	0.067	0.541	0.003	0.139	0.263
STK129509	0.048	0.963	0.989	0.5	0.795	0.046	0.089	0.668	0.004	0.557	0.969
STK129571	0.028	0.978	0.987	0.593	0.264	0.139	0.078	0.838	0.004	0.619	0.851
STK113693	0.027	0.964	0.983	0.655	0.279	0.145	0.078	0.929	0.004	0.102	0.204
STK222598	0.787	0.103	0.964	0.36	0.264	0.327	0.939	0.264	0.004	0.836	0.404
STK155936	0.111	0.11	0.982	0.033	0.652	0.055	0.116	0.795	0.003	0.048	0.24
STK222602	0.625	0.077	0.961	0.073	0.329	0.264	0.94	0.273	0.008	0.925	0.359
STK409019	0.097	0.966	0.969	0.644	0.957	0.703	0.042	0.585	0.009	0.69	0.951
STK137196	0.009	0.961	0.983	0.641	0.08	0.129	0.163	0.907	0.003	0.231	0.035
STK154090	0.03	0.985	0.985	0.684	0.171	0.079	0.077	0.884	0.004	0.621	0.731
STK130675	0.036	0.962	0.988	0.071	0.126	0.181	0.087	0.69	0.003	0.314	0.809
STK007472	0.042	0.968	0.987	0.925	0.25	0.038	0.048	0.832	0.003	0.358	0.963
STK068025	0.913	0.992	0.964	0.395	0.24	0.946	0.886	0.877	0.003	0.109	0.916
STK073398	0.02	0.973	0.988	0.278	0.305	0.029	0.066	0.813	0.004	0.907	0.841
STK075179	0.036	0.978	0.986	0.668	0.474	0.121	0.064	0.885	0.004	0.472	0.744
STK386018	0.032	0.963	0.987	0.578	0.577	0.027	0.064	0.888	0.004	0.788	0.652
STK130489	0.032	0.97	0.979	0.741	0.449	0.672	0.064	0.647	0.009	0.855	0.666
STK132568	0.008	0.956	0.988	0.856	0.03	0.028	0.192	0.915	0.003	0.365	0.063
STK401920	0.163	0.928	0.973	0.392	0.944	0.35	0.039	0.558	0.005	0.73	0.956
STK135071	0.063	0.962	0.987	0.396	0.462	0.045	0.06	0.791	0.003	0.543	0.632
STK137123	0.15	0.964	0.988	0.211	0.421	0.087	0.057	0.652	0.003	0.33	0.58
STK048780	0.061	0.928	0.987	0.209	0.518	0.042	0.083	0.741	0.004	0.841	0.685
STK031760	0.133	0.927	0.987	0.09	0.482	0.067	0.077	0.623	0.003	0.632	0.684
STK039660	0.044	0.927	0.985	0.175	0.365	0.06	0.091	0.714	0.004	0.811	0.761
STK092252	0.035	0.968	0.988	0.099	0.206	0.084	0.068	0.761	0.003	0.51	0.761
STK121703	0.619	0.226	0.964	0.432	0.231	0.551	0.936	0.325	0.004	0.868	0.085
STK325732	0.026	0.963	0.987	0.463	0.221	0.031	0.085	0.845	0.004	0.873	0.845
STK386010	0.067	0.955	0.972	0.91	0.922	0.264	0.171	0.856	0.003	0.42	0.864
STK337539	0.049	0.928	0.973	0.598	0.941	0.198	0.05	0.654	0.014	0.93	0.959
STK324799	0.013	0.967	0.978	0.925	0.338	0.085	0.113	0.853	0.005	0.786	0.954

STK337540	0.02	0.935	0.98	0.573	0.222	0.139	0.088	0.411	0.014	0.972	0.824
STK408865	0.049	0.936	0.968	0.244	0.852	0.185	0.047	0.765	0.007	0.49	0.94
STK154061	0.032	0.974	0.984	0.423	0.196	0.343	0.081	0.845	0.004	0.372	0.799
STK409037	0.048	0.971	0.978	0.79	0.548	0.638	0.049	0.729	0.007	0.761	0.456
STK409038	0.053	0.981	0.973	0.68	0.362	0.899	0.059	0.666	0.009	0.524	0.434
STK000255	0.171	0.945	0.987	0.236	0.472	0.077	0.081	0.833	0.004	0.757	0.588
STK154110	0.04	0.981	0.986	0.716	0.099	0.086	0.045	0.898	0.003	0.288	0.414
STK020405	0.05	0.962	0.985	0.592	0.843	0.384	0.326	0.618	0.004	0.361	0.306
STK401922	0.252	0.922	0.98	0.45	0.687	0.454	0.045	0.288	0.004	0.584	0.173
STK386029	0.03	0.958	0.986	0.566	0.043	0.059	0.062	0.955	0.003	0.099	0.79
STK154130	0.031	0.975	0.982	0.111	0.621	0.142	0.044	0.665	0.003	0.245	0.711
STK036626	0.134	0.917	0.977	0.3	0.448	0.621	0.05	0.225	0.005	0.57	0.131
STK097228	0.018	0.952	0.981	0.605	0.837	0.026	0.057	0.853	0.003	0.105	0.123
STK100419	0.032	0.97	0.979	0.748	0.449	0.666	0.065	0.646	0.009	0.858	0.653
STK045387	0.068	0.957	0.988	0.736	0.075	0.039	0.052	0.895	0.003	0.513	0.516
STK075062	0.023	0.944	0.985	0.214	0.032	0.038	0.079	0.922	0.003	0.132	0.756
STK122203	0.024	0.979	0.978	0.823	0.442	0.295	0.041	0.75	0.008	0.719	0.453
STK013762	0.023	0.984	0.985	0.757	0.06	0.113	0.054	0.952	0.003	0.05	0.825
STK129898	0.046	0.969	0.985	0.535	0.214	0.078	0.076	0.884	0.004	0.574	0.612
STK053591	0.028	0.943	0.986	0.505	0.046	0.059	0.1	0.943	0.004	0.28	0.788
STK188417	0.078	0.966	0.985	0.387	0.177	0.177	0.071	0.776	0.003	0.418	0.576
STK085958	0.025	0.98	0.982	0.613	0.184	0.155	0.047	0.846	0.003	0.062	0.529
STK061013	0.056	0.971	0.986	0.666	0.173	0.033	0.051	0.774	0.003	0.27	0.961
STK062148	0.132	0.983	0.986	0.985	0.458	0.334	0.351	0.927	0.004	0.638	0.922
STK081664	0.141	0.976	0.986	0.986	0.278	0.064	0.373	0.922	0.004	0.875	0.921
STK324798	0.071	0.952	0.979	0.788	0.215	0.206	0.064	0.816	0.004	0.549	0.117
STK012081	0.343	0.941	0.986	0.973	0.245	0.14	0.422	0.929	0.004	0.908	0.905
STK346841	0.628	0.443	0.983	0.012	0.279	0.701	0.132	0.359	0.003	0.156	0.314
STK044786	0.253	0.953	0.984	0.983	0.889	0.61	0.776	0.624	0.004	0.782	0.848
STK138023	0.016	0.977	0.988	0.652	0.425	0.03	0.097	0.574	0.004	0.794	0.968
STK138208	0.029	0.978	0.988	0.403	0.315	0.037	0.086	0.399	0.004	0.483	0.962

STK386019	0.045	0.966	0.986	0.754	0.062	0.03	0.07	0.898	0.003	0.484	0.853
STK133249	0.013	0.976	0.985	0.838	0.257	0.362	0.104	0.929	0.003	0.09	0.043

Table S6. Interaction with carcinogenic protein

ID	Androgen receptor	AR-LBD	Aryl hydrocarbon Receptor	Aromatase	Estrogen receptor	NR-ER-LBD	PPAR-gamma	Antioxidant Element	ATPase family AAA domain	MMP	p53
STK081237	0.611	0.014	0.949	0.934	0.849	0.027	0.004	0.499	0.359	0.807	0.061
STK280616	0.019	0.612	0.978	0.045	0.853	0.518	0.981	0.891	0.889	0.871	0.568
STK057995	0.019	0.097	0.975	0.862	0.259	0.655	0.952	0.884	0.806	0.86	0.82
STK408850	0.022	0.556	0.974	0.116	0.578	0.124	0.967	0.704	0.795	0.794	0.616
STK067256	0.325	0.345	0.977	0.539	0.732	0.429	0.984	0.922	0.958	0.931	0.747
STK100429	0.025	0.075	0.755	0.821	0.776	0.095	0.983	0.892	0.555	0.952	0.89
STK362117	0.624	0.312	0.988	0.861	0.961	0.796	0.765	0.953	0.961	0.977	0.792
STK387431	0.014	0.749	0.97	0.148	0.798	0.48	0.975	0.889	0.849	0.939	0.726
STK046443	0.509	0.004	0.806	0.063	0.183	0.004	0.002	0.603	0.025	0.357	0.079
STK136267	0.641	0.125	0.813	0.033	0.834	0.005	0.935	0.722	0.947	0.73	0.377
STK386021	0.005	0.078	0.872	0.102	0.893	0.013	0.968	0.822	0.71	0.914	0.899
STK137950	0.518	0.183	0.883	0.178	0.861	0.011	0.96	0.667	0.92	0.865	0.484
STK385466	0.038	0.04	0.899	0.88	0.708	0.015	0.951	0.746	0.687	0.93	0.795
STK385674	0.473	0.024	0.905	0.857	0.917	0.073	0.926	0.904	0.931	0.945	0.708
STK072483	0.544	0.104	0.89	0.658	0.918	0.137	0.939	0.849	0.933	0.909	0.427
STK154114	0.59	0.085	0.905	0.343	0.878	0.017	0.871	0.866	0.934	0.901	0.731
STK012551	0.567	0.074	0.912	0.321	0.912	0.027	0.93	0.801	0.916	0.908	0.353
STK131655	0.037	0.044	0.762	0.063	0.572	0.005	0.862	0.634	0.838	0.726	0.43
STK154089	0.589	0.317	0.883	0.338	0.787	0.012	0.952	0.712	0.944	0.796	0.534
STK129297	0.006	0.034	0.882	0.623	0.887	0.016	0.968	0.882	0.698	0.939	0.918
STK129615	0.501	0.059	0.836	0.349	0.875	0.016	0.95	0.695	0.926	0.856	0.364
STK090091	0.396	0.253	0.942	0.92	0.906	0.706	0.951	0.943	0.918	0.973	0.909
STK129509	0.32	0.009	0.823	0.519	0.529	0.008	0.875	0.784	0.953	0.674	0.481
STK129571	0.016	0.074	0.869	0.478	0.679	0.009	0.932	0.516	0.765	0.859	0.568
STK113693	0.015	0.025	0.949	0.835	0.279	0.018	0.766	0.773	0.516	0.607	0.444
STK222598	0.041	0.01	0.977	0.929	0.892	0.205	0.319	0.93	0.779	0.97	0.82
STK155936	0.064	0.074	0.921	0.773	0.886	0.026	0.012	0.844	0.773	0.909	0.489
STK222602	0.049	0.008	0.981	0.914	0.888	0.019	0.156	0.9	0.746	0.967	0.79

STK409019	0.049	0.008	0.827	0.082	0.649	0.006	0.607	0.678	0.674	0.501	0.366
STK137196	0.01	0.008	0.746	0.022	0.716	0.006	0.668	0.307	0.072	0.687	0.255
STK154090	0.625	0.22	0.863	0.177	0.864	0.014	0.894	0.757	0.94	0.819	0.551
STK130675	0.425	0.39	0.935	0.895	0.914	0.584	0.948	0.92	0.924	0.95	0.841
STK007472	0.107	0.68	0.925	0.771	0.958	0.239	0.981	0.936	0.938	0.954	0.842
STK068025	0.266	0.002	0.846	0.031	0.131	0.003	0.002	0.166	0.315	0.256	0.012
STK073398	0.571	0.258	0.917	0.651	0.89	0.048	0.951	0.781	0.918	0.909	0.519
STK075179	0.017	0.023	0.786	0.663	0.769	0.013	0.912	0.558	0.791	0.848	0.541
STK386018	0.364	0.014	0.818	0.556	0.929	0.06	0.86	0.806	0.936	0.882	0.253
STK130489	0.011	0.012	0.898	0.583	0.751	0.009	0.8	0.555	0.552	0.678	0.566
STK132568	0.008	0.076	0.806	0.172	0.741	0.011	0.736	0.573	0.262	0.698	0.683
STK401920	0.658	0.006	0.863	0.195	0.916	0.012	0.507	0.868	0.944	0.723	0.515
STK135071	0.36	0.015	0.855	0.794	0.928	0.075	0.889	0.89	0.926	0.924	0.654
STK137123	0.311	0.016	0.882	0.883	0.925	0.111	0.913	0.915	0.908	0.953	0.864
STK048780	0.056	0.01	0.858	0.579	0.884	0.02	0.823	0.737	0.91	0.921	0.649
STK031760	0.059	0.011	0.88	0.8	0.884	0.024	0.843	0.856	0.888	0.952	0.829
STK039660	0.056	0.008	0.883	0.224	0.875	0.009	0.798	0.626	0.894	0.918	0.607
STK092252	0.555	0.303	0.93	0.882	0.901	0.435	0.927	0.911	0.927	0.947	0.778
STK121703	0.149	0.011	0.977	0.94	0.885	0.783	0.239	0.934	0.822	0.977	0.822
STK325732	0.382	0.048	0.884	0.331	0.925	0.036	0.908	0.815	0.931	0.891	0.394
STK386010	0.039	0.139	0.875	0.603	0.937	0.007	0.764	0.942	0.886	0.841	0.752
STK337539	0.666	0.015	0.756	0.027	0.919	0.006	0.571	0.825	0.931	0.56	0.229
STK324799	0.28	0.059	0.952	0.878	0.949	0.273	0.892	0.898	0.962	0.918	0.877
STK337540	0.538	0.021	0.912	0.236	0.936	0.03	0.801	0.761	0.932	0.747	0.409
STK408865	0.217	0.005	0.881	0.493	0.771	0.006	0.16	0.725	0.829	0.737	0.393
STK154061	0.021	0.016	0.83	0.123	0.687	0.005	0.895	0.492	0.581	0.852	0.471
STK409037	0.009	0.006	0.843	0.732	0.807	0.013	0.757	0.586	0.625	0.685	0.455
STK409038	0.003	0.006	0.863	0.596	0.313	0.007	0.797	0.559	0.026	0.632	0.408

STK000255	0.082	0.035	0.849	0.77	0.884	0.063	0.75	0.848	0.935	0.921	0.751
STK154110	0.383	0.492	0.865	0.521	0.857	0.016	0.955	0.788	0.867	0.872	0.677
STK020405	0.232	0.005	0.932	0.942	0.781	0.388	0.192	0.762	0.934	0.591	0.531
STK401922	0.457	0.005	0.898	0.891	0.928	0.231	0.751	0.903	0.936	0.921	0.809
STK386029	0.613	0.392	0.954	0.359	0.941	0.338	0.661	0.9	0.97	0.871	0.828
STK154130	0.038	0.024	0.435	0.88	0.878	0.178	0.965	0.737	0.363	0.944	0.369
STK036626	0.498	0.004	0.907	0.848	0.92	0.036	0.7	0.892	0.911	0.922	0.767
STK097228	0.018	0.013	0.745	0.034	0.44	0.009	0.508	0.546	0.075	0.339	0.085
STK100419	0.011	0.012	0.895	0.552	0.737	0.009	0.811	0.579	0.549	0.684	0.553
STK045387	0.206	0.332	0.867	0.83	0.93	0.324	0.936	0.895	0.897	0.904	0.712
STK075062	0.615	0.499	0.956	0.305	0.89	0.075	0.91	0.92	0.964	0.908	0.87
STK122203	0.469	0.009	0.881	0.71	0.89	0.019	0.871	0.629	0.941	0.682	0.314
STK013762	0.588	0.546	0.942	0.39	0.858	0.045	0.786	0.859	0.965	0.859	0.867
STK129898	0.593	0.056	0.875	0.433	0.925	0.094	0.767	0.835	0.941	0.865	0.502
STK053591	0.598	0.343	0.956	0.148	0.881	0.022	0.785	0.871	0.968	0.91	0.867
STK188417	0.588	0.061	0.885	0.674	0.922	0.084	0.774	0.892	0.938	0.912	0.793
STK085958	0.06	0.076	0.861	0.686	0.429	0.011	0.723	0.618	0.818	0.712	0.655
STK061013	0.25	0.587	0.938	0.836	0.973	0.314	0.985	0.95	0.968	0.968	0.903
STK062148	0.02	0.446	0.833	0.88	0.655	0.027	0.905	0.774	0.614	0.929	0.69
STK081664	0.124	0.323	0.87	0.847	0.914	0.192	0.925	0.882	0.867	0.941	0.735
STK324798	0.162	0.008	0.905	0.928	0.91	0.454	0.246	0.865	0.917	0.866	0.774
STK012081	0.021	0.206	0.875	0.849	0.813	0.062	0.851	0.87	0.825	0.956	0.802
STK346841	0.192	0.046	0.98	0.96	0.93	0.133	0.914	0.96	0.993	0.919	0.929
STK044786	0.071	0.02	0.909	0.904	0.815	0.055	0.663	0.839	0.793	0.853	0.704
STK138023	0.761	0.028	0.906	0.062	0.956	0.025	0.971	0.904	0.973	0.938	0.385
STK138208	0.736	0.034	0.914	0.209	0.957	0.029	0.973	0.926	0.969	0.958	0.763
STK386019	0.505	0.266	0.927	0.416	0.881	0.024	0.933	0.845	0.938	0.88	0.591
STK133249	0.005	0.012	0.703	0.155	0.329	0.007	0.76	0.456	0.023	0.655	0.48
