# Supplementary material for: Pharmacophore-based virtual screening of commercial databases against β-secretase 1 for drug development against Alzheimer’s disease
Source: Front Chem. 2024 Jul 9;12:1412349. doi: 10.3389/fchem.2024.1412349 (PMC11263123; doi:10.3389/fchem.2024.1412349)
Supplement: Supplementary file 2 [file DataSheet1.PDF]

Suppletory figures. Molecular structures of all the 84 compounds.

S. No.	Compound ID	Molecular structure
1	STK081237	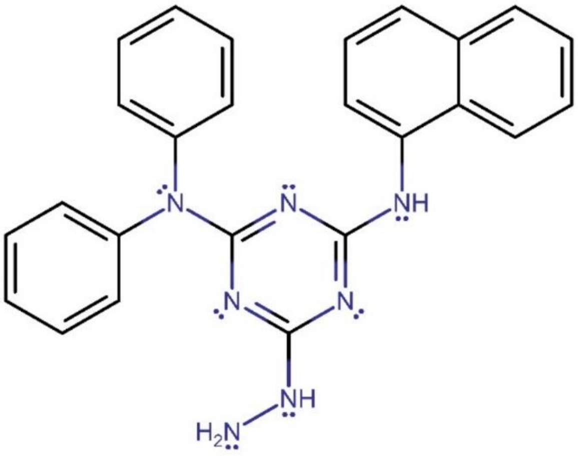
2	STK280616	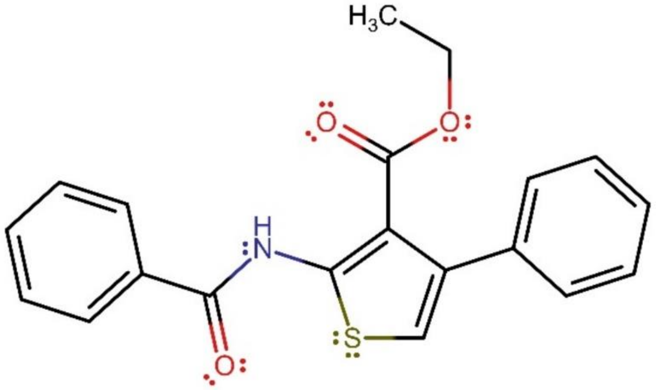
3	STK057995	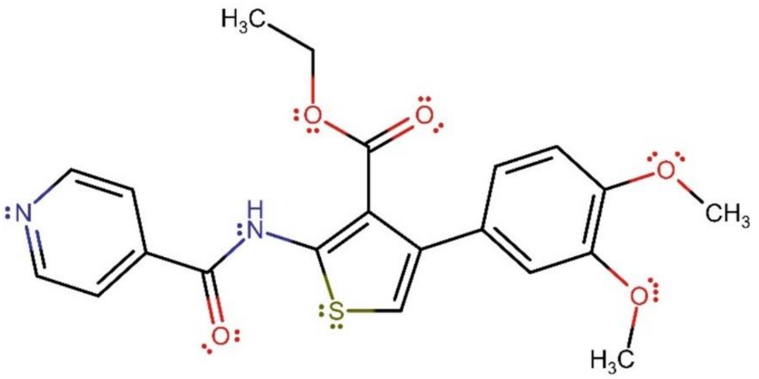

4	STK408850	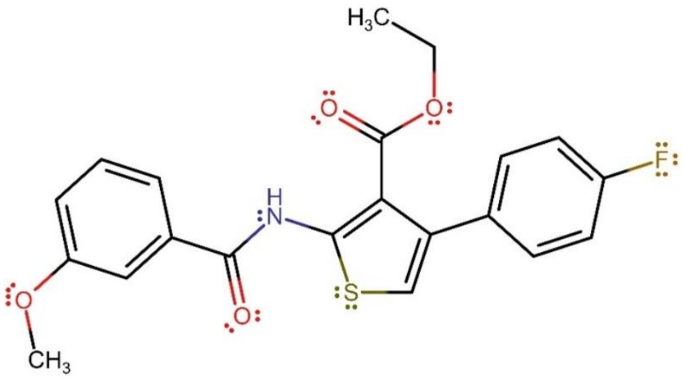
5	STK067256	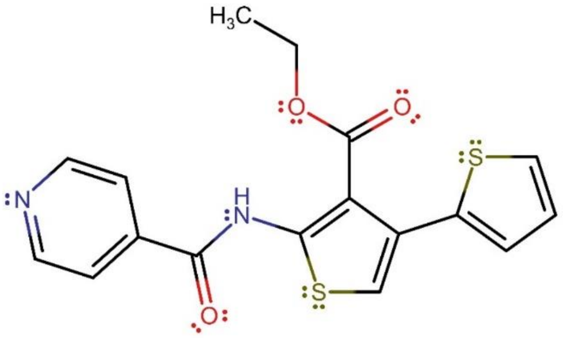
6	STK100429	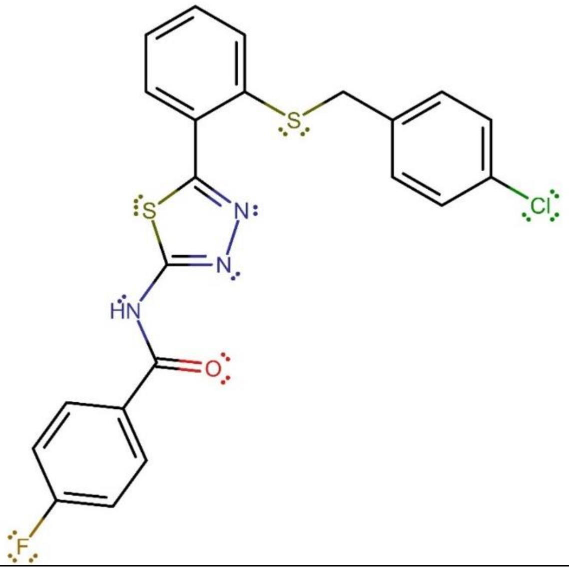

7	STK362117	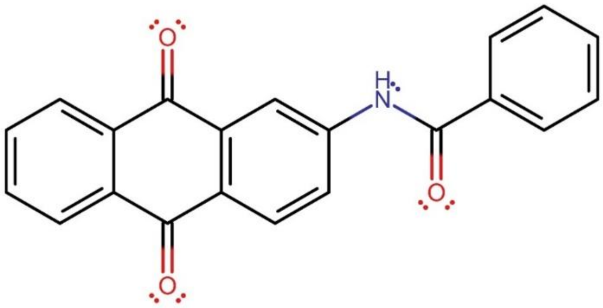
8	STK387431	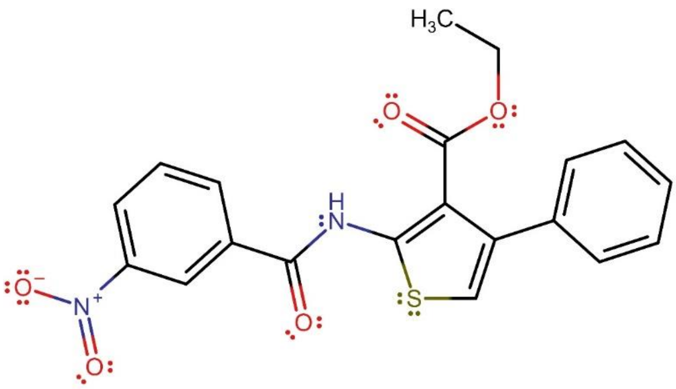

9	STK046443	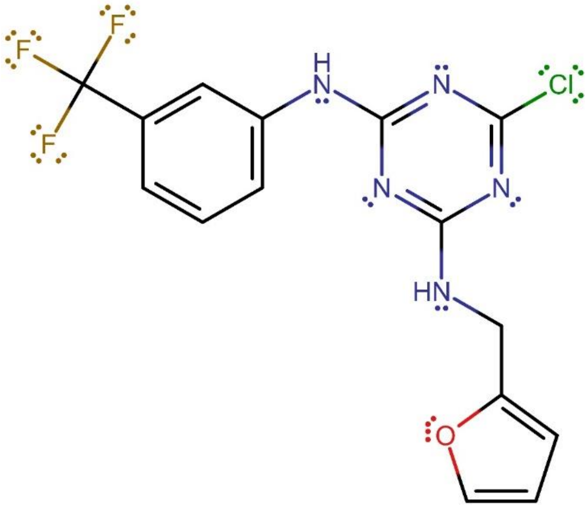
10	STK136267	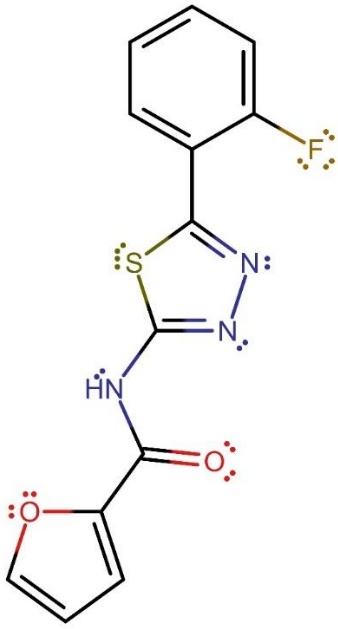

11	STK386021	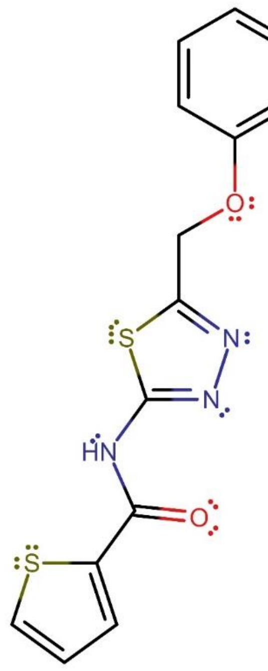
12	STK137950	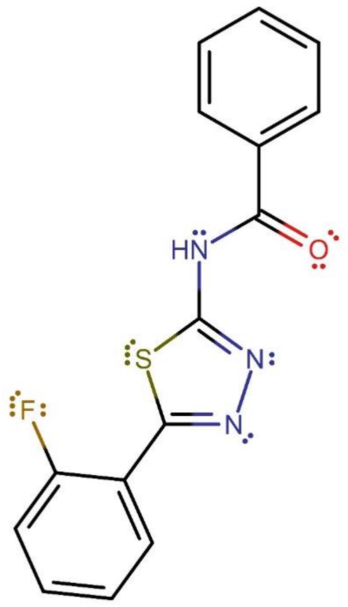

13	STK385466	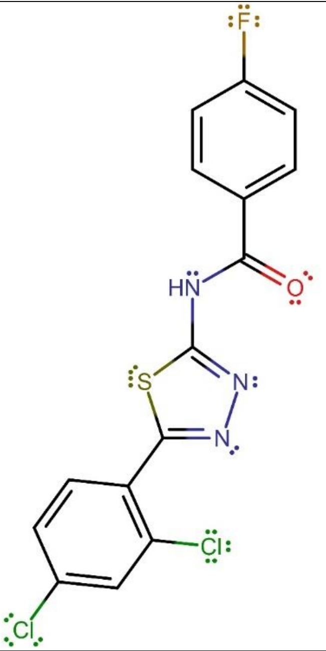
14	STK385674	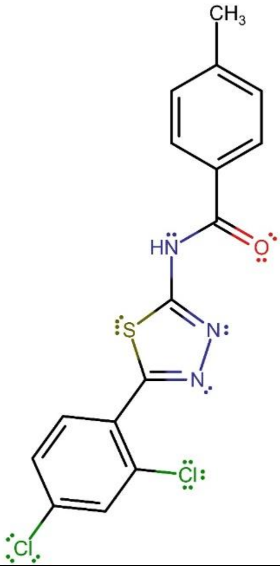
15	STK072483	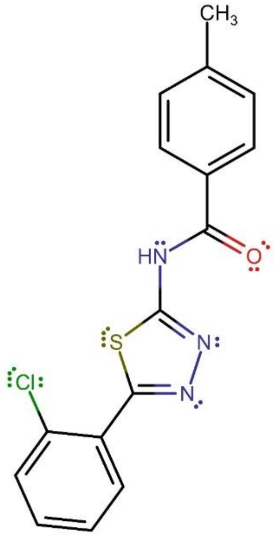

16	STK154114	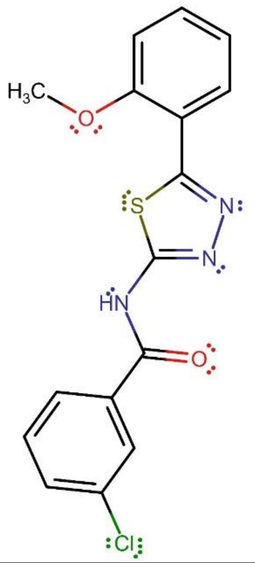
17	STK012551	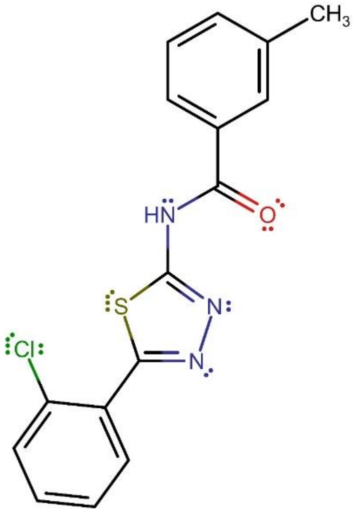
18	STK131655	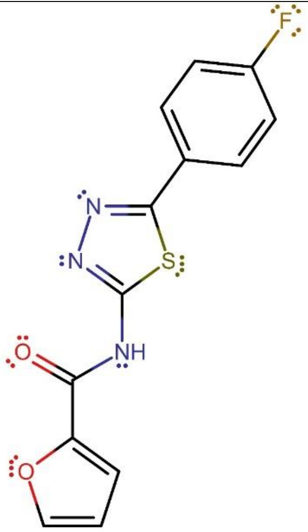

19	STK154089	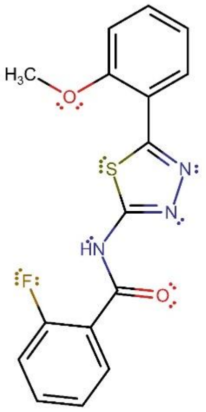
20	STK129297	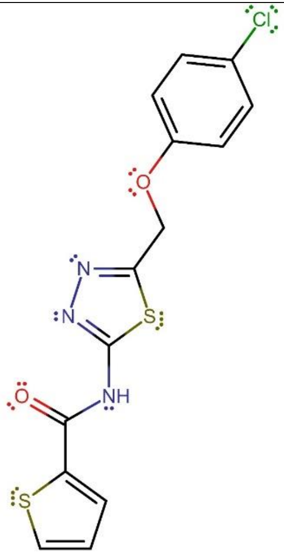
21	STK129615	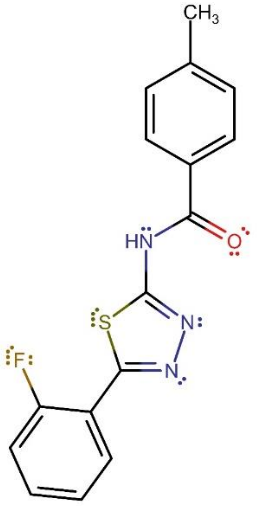

22	STK090091	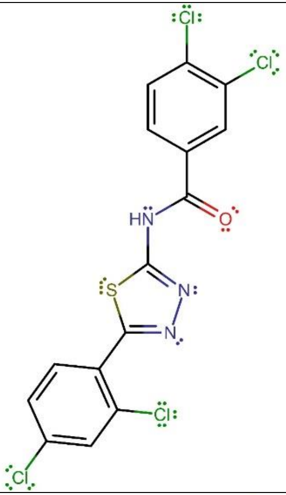
23	STK129509	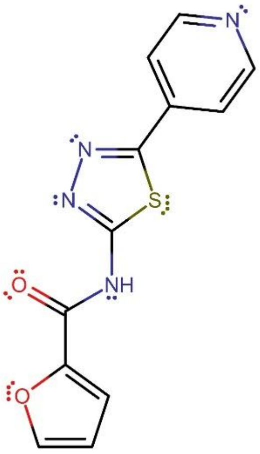
24	STK129571	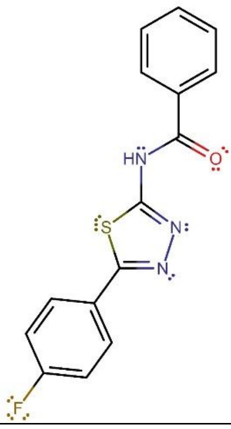

25	STK113693	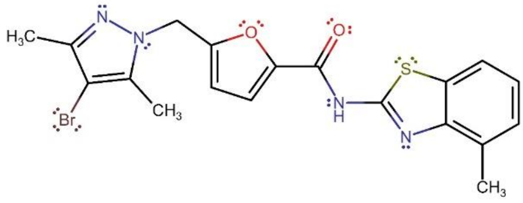
26	STK222598	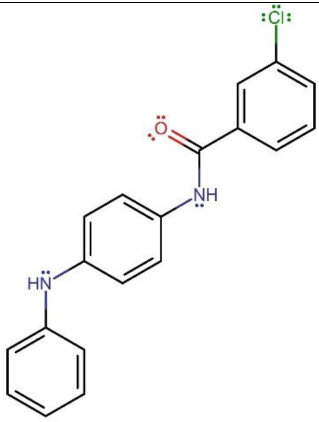
27	STK155936	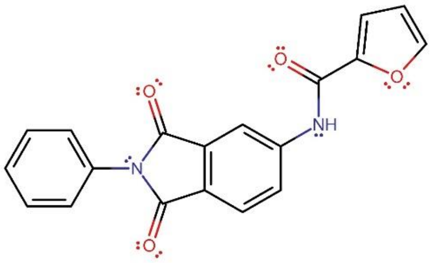

28	STK222602	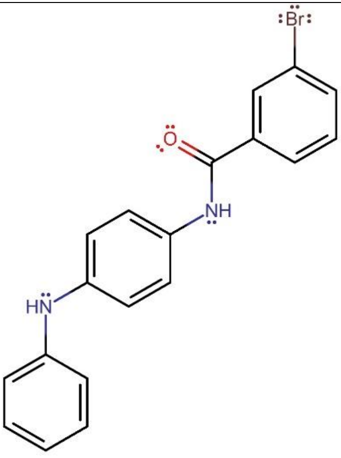
29	STK409019	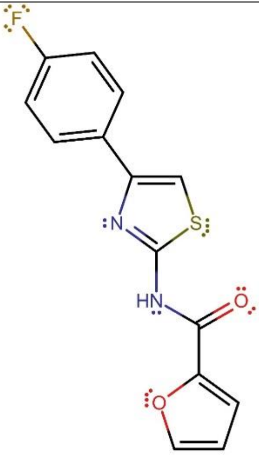
30	STK137196	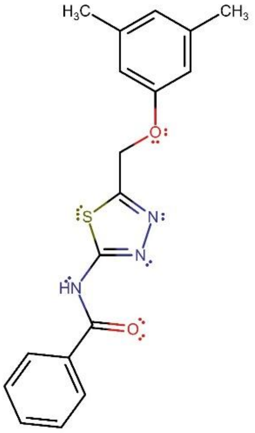

31	STK154090	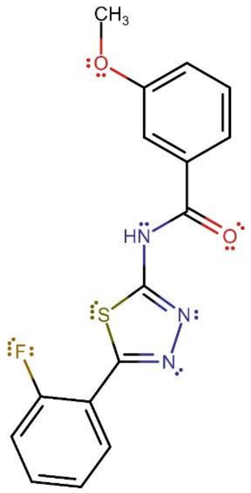
32	STK130675	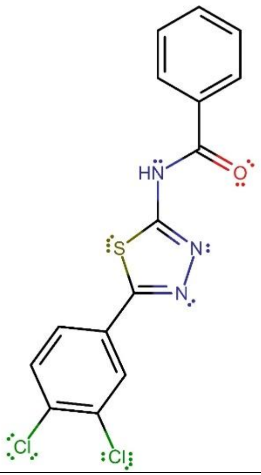
33	STK007472	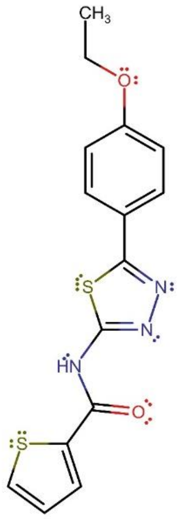

34	STK068025	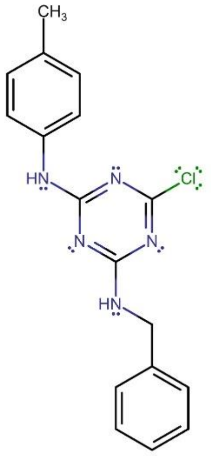
35	STK073398	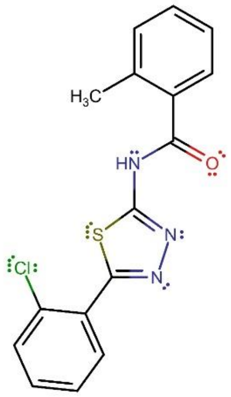
36	STK075179	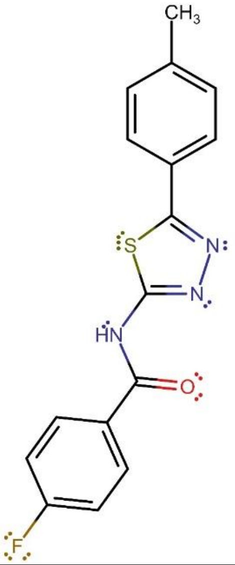

37	STK386018	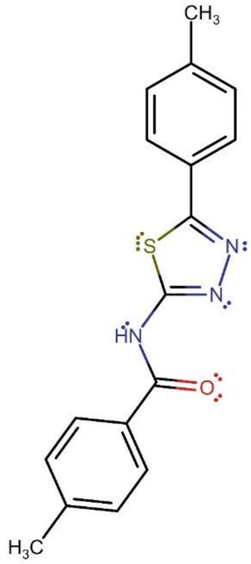
38	STK130489	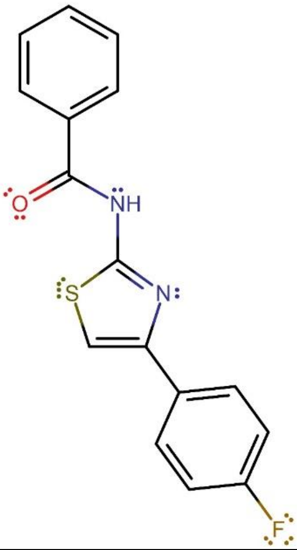
39	STK132568	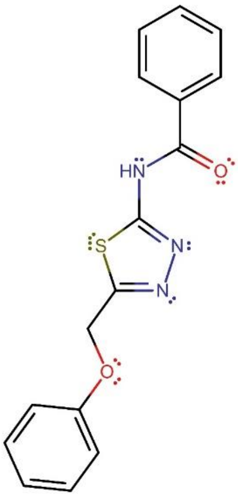

40	STK401920	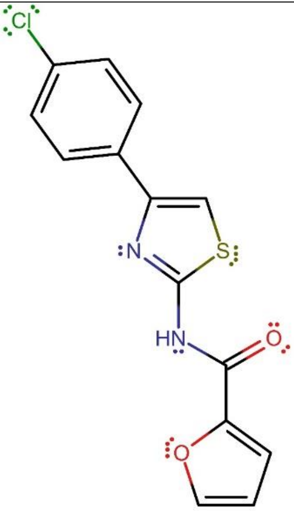
41	STK135071	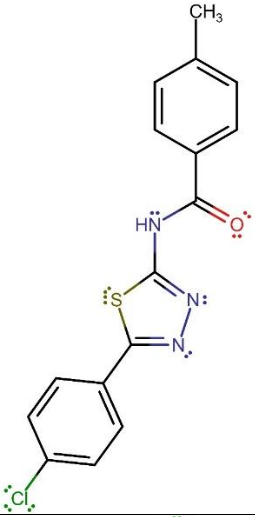
42	STK137123	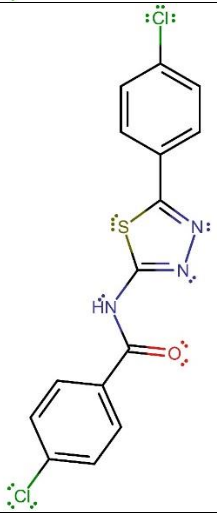

43	STK048780	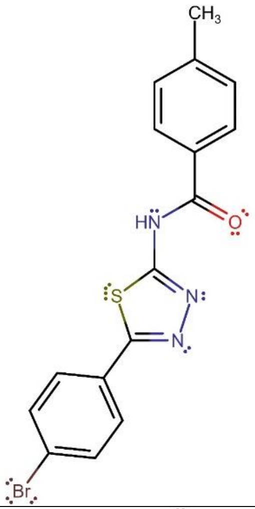
44	STK031760	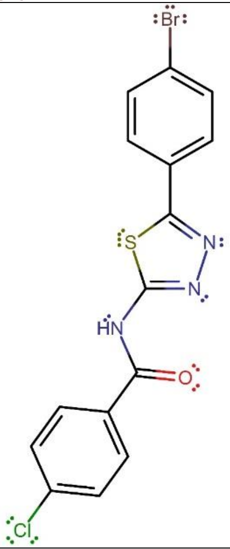
45	STK039660	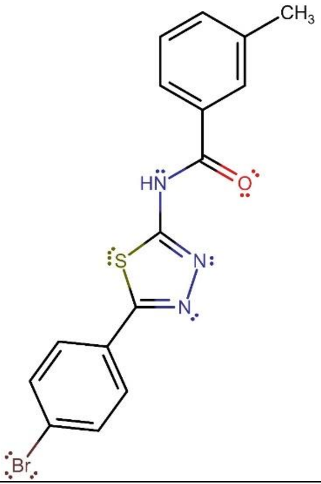

46	STK092252	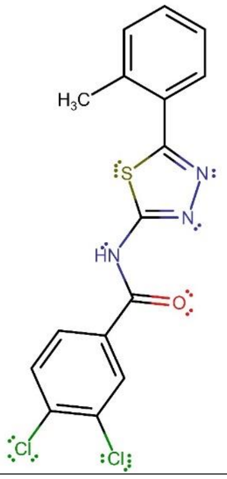
47	STK121703	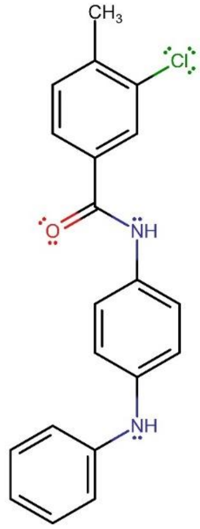
48	STK325732	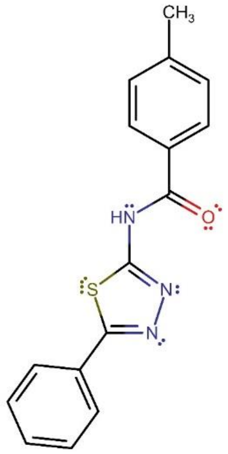

49	STK386010	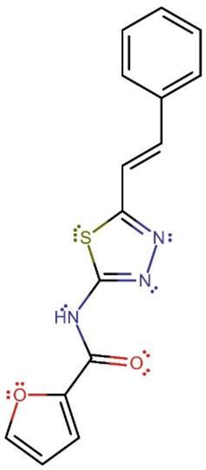
50	STK337539	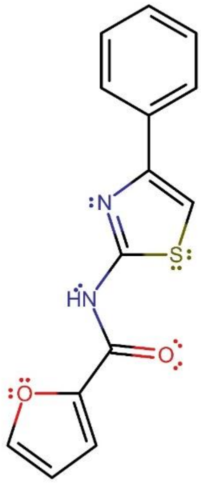
51	STK324799	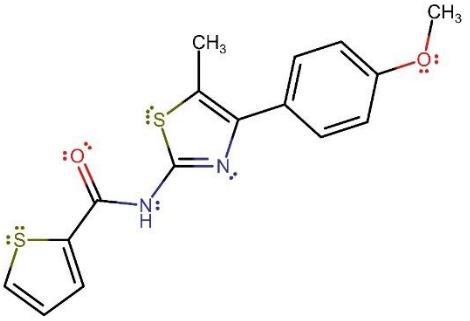

52	STK337540	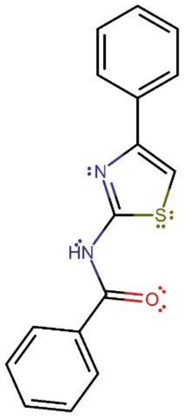
53	STK408865	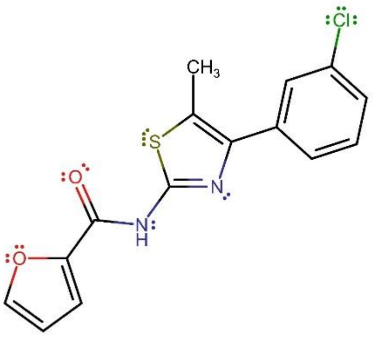
54	STK154061	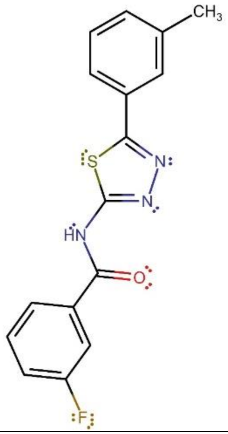
55	STK409037	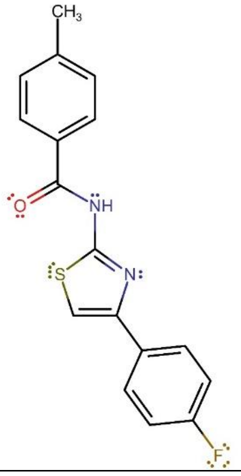

56	STK409038	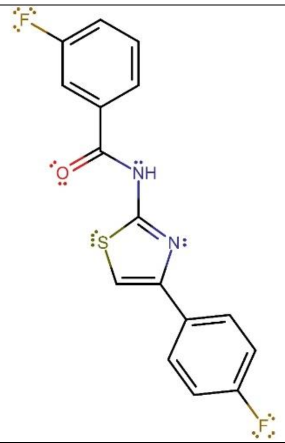
57	STK000255	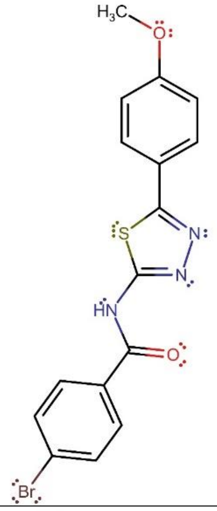
58	STK154110	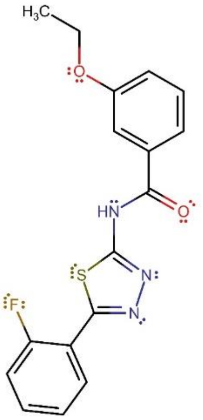
59	STK020405	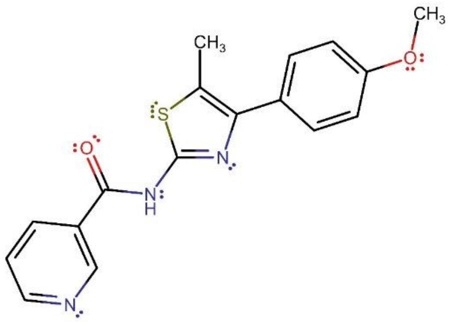

60	STK401922	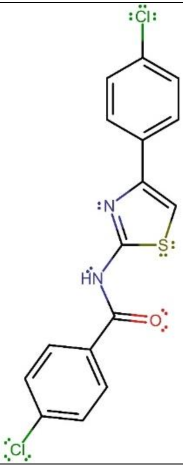
61	STK386029	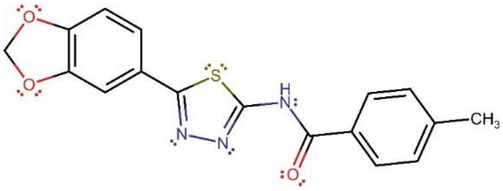
62	STK154130	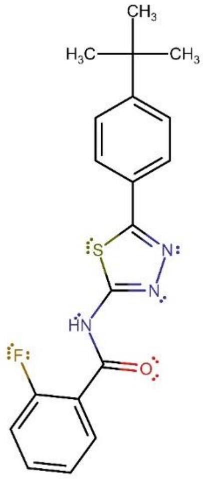

63	STK036626	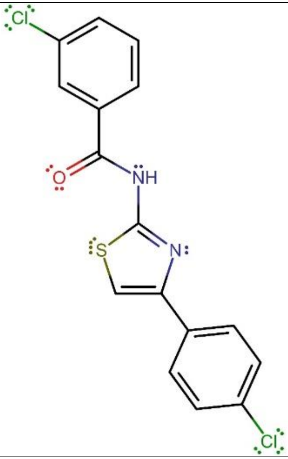
64	STK097228	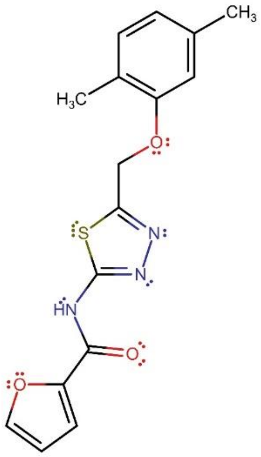
65	STK100419	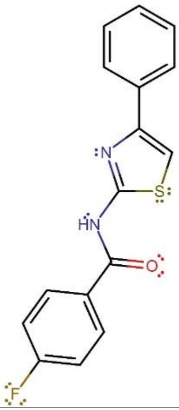
66	STK045387	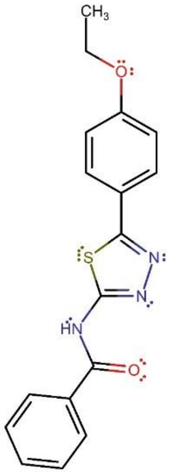

67	STK075062	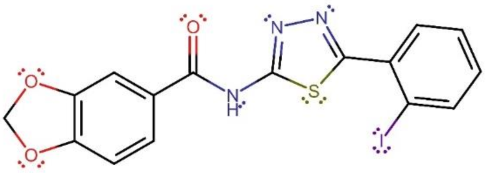
68	STK122203	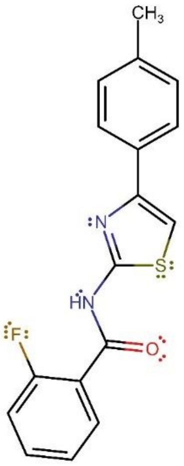
69	STK013762	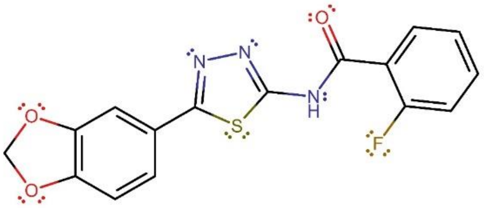

70	STK129898	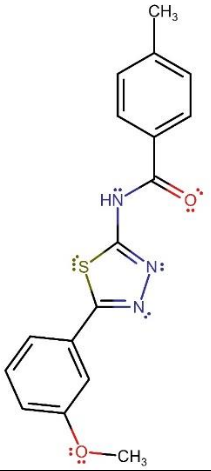
71	STK053591	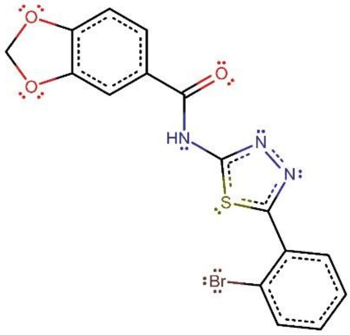
72	STK188417	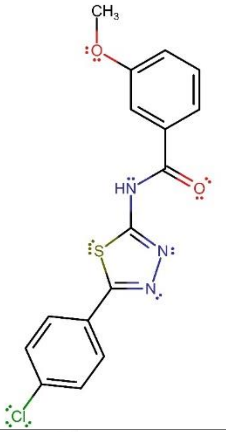
73	STK085958	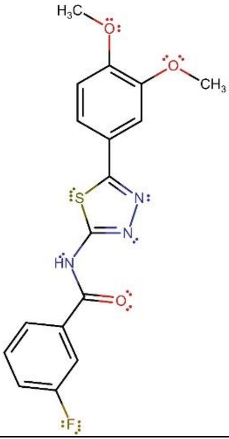

74	STK061013	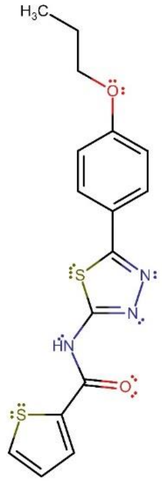
75	STK062148	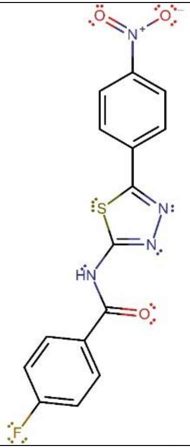
76	STK081664	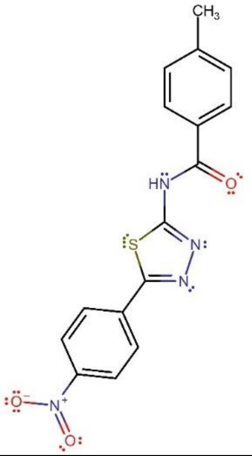
77	STK324798	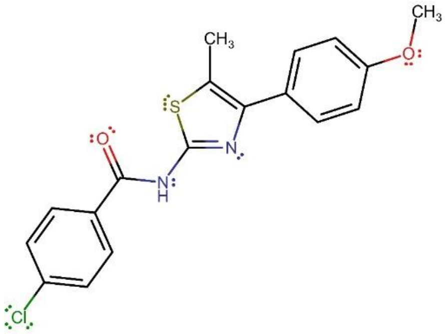

78	STK012081	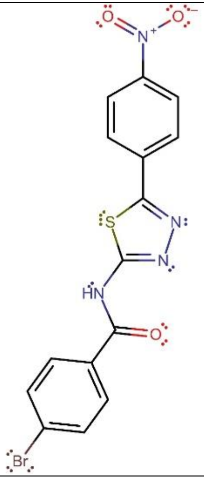
79	STK346841	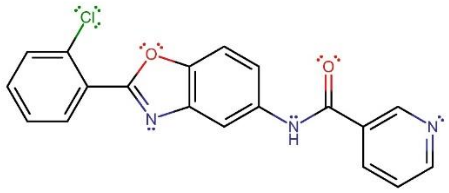
80	STK044786	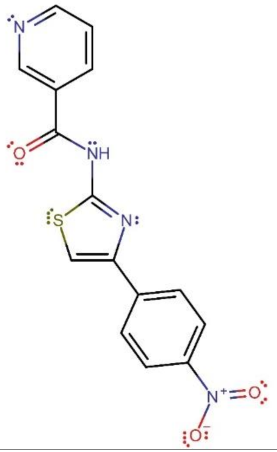
81	STK138023	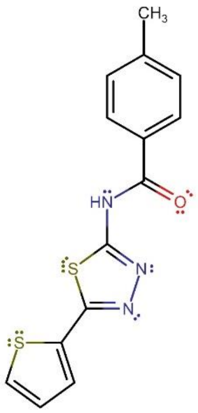

82	STK138208	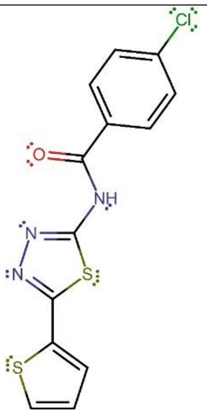
83	STK386019	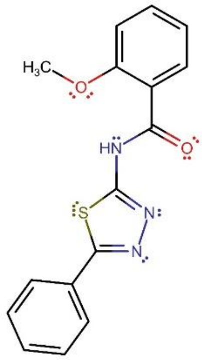
84	STK133249	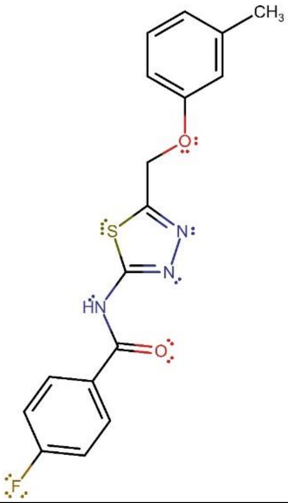
